# Screening for modulators of the cellular composition of gut epithelia via organoid models of intestinal stem cell differentiation

**DOI:** 10.1038/s41551-022-00863-9

**Published:** 2022-03-21

**Authors:** Benjamin E. Mead, Kazuki Hattori, Lauren Levy, Shinya Imada, Norihiro Goto, Marko Vukovic, Daphne Sze, Conner Kummerlowe, Juan D. Matute, Jinzhi Duan, Robert Langer, Richard S. Blumberg, Jose Ordovas-Montanes, Ömer H. Yilmaz, Jeffrey M. Karp, Alex K. Shalek

**Affiliations:** 1 Harvard-MIT Program in Health Sciences and Technology, MIT, Cambridge, MA USA; 2 Koch Institute for Integrative Cancer Research, MIT, Cambridge, MA USA; 3 Harvard Stem Cell Institute, Cambridge, MA USA; 4 Broad Institute of MIT and Harvard, Cambridge, MA USA; 5 Institute for Medical Engineering and Science, MIT, Cambridge, MA USA; 6 Department of Chemistry, MIT, Cambridge, MA USA; 7 Ragon Institute of MGH, MIT and Harvard, Cambridge, MA USA; 8 Engineering in Medicine, Department of Medicine, Brigham and Women’s Hospital, Harvard Medical School, Boston, MA USA; 9 Division of Gastroenterology Boston Children’s Hospital, Program in Immunology, Harvard Medical School, Boston, MA USA; 10 Division of Gastroenterology, Department of Medicine, Brigham and Women’s Hospital, Harvard Medical School, Boston, MA USA; 11 Division of Neonatology, Department of Pediatrics, MGH Harvard Medical School, Boston, MA USA; 12 Department of Chemical Engineering, MIT, Cambridge, MA USA; 13 Department of Pathology, MGH, Harvard Medical School, Boston, MA USA

**Keywords:** Intestinal stem cells, Systems biology, Tissue engineering, High-throughput screening

## Abstract

The cellular composition of barrier epithelia is essential to organismal homoeostasis. In particular, within the small intestine, adult stem cells establish tissue cellularity, and may provide a means to control the abundance and quality of specialized epithelial cells. Yet, methods for the identification of biological targets regulating epithelial composition and function, and of small molecules modulating them, are lacking. Here we show that druggable biological targets and small-molecule regulators of intestinal stem cell differentiation can be identified via multiplexed phenotypic screening using thousands of miniaturized organoid models of intestinal stem cell differentiation into Paneth cells, and validated via longitudinal single-cell RNA-sequencing. We found that inhibitors of the nuclear exporter Exportin 1 modulate the fate of intestinal stem cells, independently of known differentiation cues, significantly increasing the abundance of Paneth cells in the organoids and in wild-type mice. Physiological organoid models of the differentiation of intestinal stem cells could find broader utility for the screening of biological targets and small molecules that can modulate the composition and function of other barrier epithelia.

## Abstract (web summary)

Organoid models of intestinal stem cell differentiation into Paneth cells allow for the identification, via high-throughput phenotypic screening, of biological targets and small molecules regulating the composition of intestinal epithelium.

## Main

Barrier tissues interact with the external environment and protect from it. These vital functions are accomplished by specialized epithelial cells, descendant from epithelial stem cells, and are supported by stromal and immune cell populations. Balanced cellular composition in these barrier tissues is critical for host health. In the upper respiratory tract and skin, for example, changes in epithelial cellularity arising from aberrant stem cell differentiation can precipitate inflammatory diseases^1,2^. Similarly, shifts in the composition and quality of mature epithelial cells are known to occur in the colon and small intestine of patients suffering from inflammatory bowel disease^3^. Cellular differentiation from the intestinal stem cell (ISC) niche is fluid and responsive to both physiologic and pathologic stimuli^4^, and ISCs have a capacity to integrate dietary and immune-derived signals to modulate their self-renewal and differentiation into specific secretory lineages^5–7^. Given this plasticity and the importance of barrier cellularity, barrier stem cells are a compelling target for therapeutic development.

To support discovery efforts to modulate epithelial barriers across the spectrum of health and disease, there is a need to scale approaches that can identify druggable biological targets that regulate epithelial composition and function. Ideally, these efforts would take place as close to the patient as possible. However, testing in vivo is poorly scaled and complicated by ethical boundaries. High-throughput testing is possible in cell lines, but they are limited by poor representation of tissue-level biology, which can hinder translation^8^. To empower true target identification, we require in vitro cellular models that faithfully recapitulate the barrier tissue cells and functional processes that occur in vivo.

Modelling of epithelial barrier tissues, such as the intestine, in vitro has advanced substantially over the past decade thanks to the widespread development of organoid models. Intestinal organoids—broadly defined as three-dimensional (3D), stem cell-derived, tissue-like cellular structures—have proven to be valuable models of the adult stem cell niche, and preserve known developmental pathways in stem cell differentiation^9,10^. The addition of well-characterized small molecules to culture media enables intestinal organoids to be further enriched for ISCs and can also be used to drive differentiation down specific lineages via physiologically meaningful cues, such as the modulation of Wnt and Notch signalling pathways^10^. Use of such rationally directed differentiation has been applied to induce functional Paneth cells (an antimicrobial-producing cell of the small intestinal crypt and proximal colon in humans) from enriched ISCs in vitro^11^. Further, foundational work with murine intestinal organoids has illuminated the intricacies of how these multicellular systems initially self-assemble^12^, providing an insightful landscape into the phenotypic states accessible to these models^13^.

Yet, organoid models are dynamic, cellularly and structurally heterogeneous, and typically require complex and costly experimental manipulations. This has limited their application as a screening tool to inform in vivo tissue biology. To date, organoids have primarily been used at scale to either decipher fundamentals of organoid biology^13,14^ or in the context of malignancy where the therapeutic phenotype (for example, growth inhibition) is easily measured^15–17^. Thus, while such work has been foundational towards harnessing organoids for screening, it has not yet yielded a scalable start-to-finish discovery pipeline to identify tissue-modifying agents that operate from initial discovery to in vivo validation.

We sought to test whether a framework utilizing organoid models could be used to identify translatable barrier tissue-modifying small molecules. Broadly, such a framework can be described in 4 steps: (1) choosing a specific physiological process that is well-modelled by an organoid and perform a phenotypic screen for marker(s) of desired effect; (2) prioritizing lead compound(s) through a rigorous statistical approach and validating compound(s) in orthogonal assays; (3) exploring compound-mediated biology in organoid model with a high-content assay (for example, single-cell RNA-seq) to examine putative mechanism of action; and (4) where cellular mechanisms dictate potential for translation, testing select compound(s) in vivo to validate intended effect.

More specifically, we aimed to screen for pharmaceutically actionable biological targets that mediate a physiological differentiation process independent of major niche-associated pathways. We adapted organoids for phenotypic high-throughput screening through the reduction of model complexity around a well-structured hypothesis – here, to modulate physiological Paneth cell differentiation – that incorporates links to in vivo tissue biology^18,19^. Searching for unreported targets that enhance Paneth cell differentiation and increase their abundance in the native tissue may be therapeutically valuable. Declines in Paneth cell quality and number are observed in inflammatory bowel disease^20–22^, necrotizing enterocolitis^23^, environmental enteric dysfunction^24^ and intestinal manifestations of graft versus host disease (GvHD)^25^. Additionally, treatment with R-spondin 1, a potentiator of Wnt signalling, can resolve dysbiosis seen in mice with GvHD by stimulating ISCs to differentiate into Paneth cells^26^, but is challenging to apply clinically as Wnt activation is implicated in precancerous hyperplasia^27^. Other signalling pathways known to drive Paneth cell differentiation, including Notch signalling, face similar challenges^28^.

Through the development and application of a screening framework, we identify previously unreported targets and associated agents that may meaningfully enhance in vivo Paneth cell abundance. Following initial screening efforts, we perform robust cross-species in vitro investigation of our most potent hit to identify a key lead and its underlying biology. From there, we demonstrate translation in a murine model, showing a specific increase in Paneth cell abundance in vivo. Overall, our work defines an extendable paradigm with which to discover targets and their cognate pharmacophores for rationally modulating epithelial barrier cellularity.

## Results

**Fig. 1:**
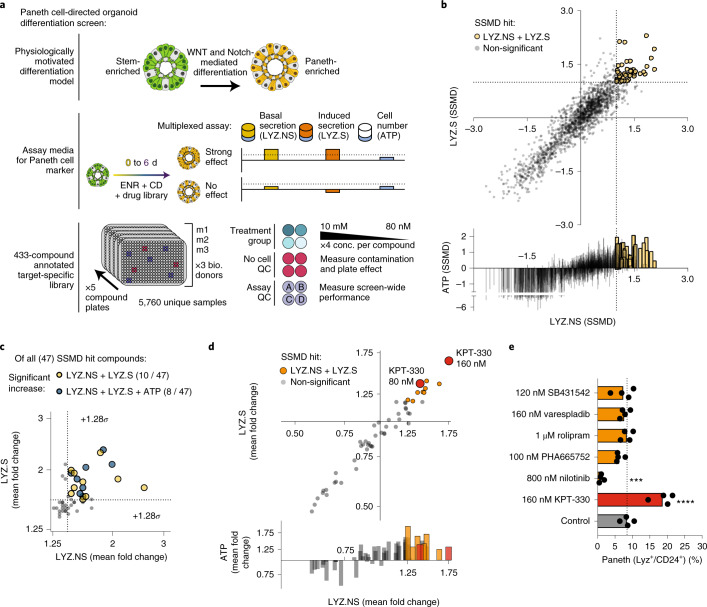
High-throughput organoid differentiation screen reveals pro-Paneth function compounds. **a**, Stem-enriched to Paneth-enriched (ENR+CD) organoid differentiation-modulating small-molecule screen (top row) assayed with multiplexed functional secretion, both basal (LYZ.NS) and 10 uM carbachol-stimulated (induced) lysozyme (LYZ.S) secretion, and cell number (ATP) on day 6 (middle row). Screened compounds (433) dosed at 4 concentrations (80 nM to 10 mM) were randomly distributed across 5 plates with interspersed quality control (QC) wells including both no cell and stimulated and non-stimulated (A,B,C,D) (bottom row), with the full screen repeated with organoids derived from 3 murine donors (m1–m3). **b**, Replicate strictly standardized mean difference (SSMD) for each assay in the primary screen, each point representing the SSMD from 3 replicates of 3 biological donors relative to the whole-plate control. Coloured points are hits above the false positive limit- and false negative limit-determined cutoff (dotted lines) in both LYZ.NS and LYZ.S assays. **c**, Mean fold change of assay effect for hits in LYZ.S and LYZ.NS (yellow) or all three assays (blue) in the primary screen; only points above 1.28 s.d. (*σ*) (dotted lines) of all treatment mean fold changes for LYZ.S and LYZ.NS are deemed significantly increased. **d**, Mean fold change for each assay in the secondary validation screen (*N* = 8 well replicates, relative to DMSO controls); orange, treatments advanced for profiling; red, most potent compound, KPT-330. **e**, Flow cytometry for the mature Paneth cell fraction of all live cells in 3D-cultured intestinal organoids treated with 6 hit compounds during 6 d of culture in ENR+CD media. Paneth cells were identified as lysozyme-positive and CD24-mid cells. Means and individual values are shown (*N* = 4); dotted line represents the average Paneth cell fraction in control samples. One-way analysis of variance (ANOVA) post-hoc Dunnett’s multiple comparisons test: ****P < 0.0001, ***P = 0.001. Source data

**Extended Data Fig. 1:**
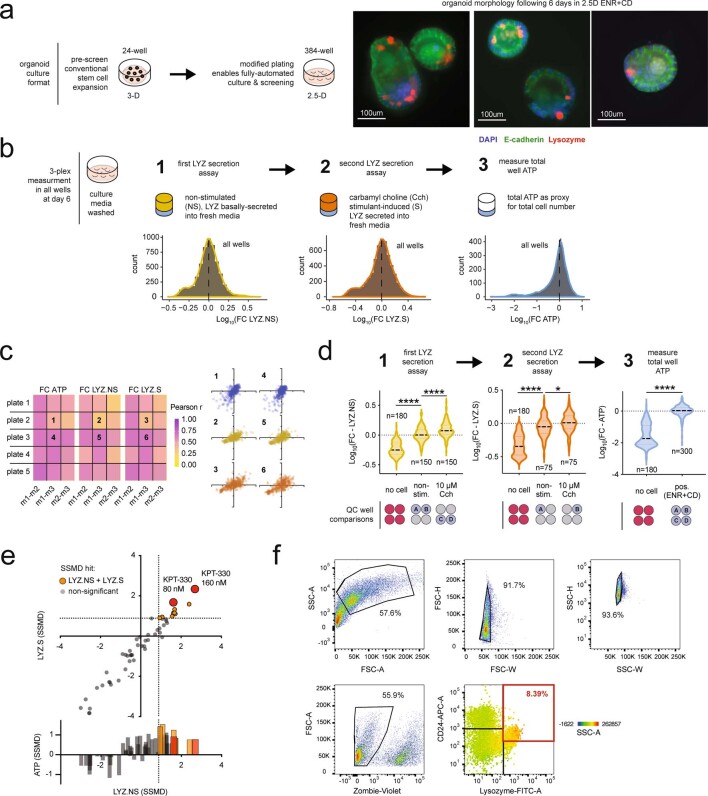
Characterization of small intestinal organoid screening approach. **a**, Organoids grown in stem-enriched media and transferred to culture in 2.5D system for 6 d (ENR + CD), Paneth cells marked by Lysozyme, representative image of 3 from single experiment shown. **b**, Tri-plex assay order and distributions of all sample data (N = 5676 wells) for each assay (numbered by order of assay) following data transformation and normalization, dotted line indicates median of distribution from which fold change calculations are determined. **c**, Pearson correlation (r) between all sample wells by screen plate and biological replicate (m1, m2, m3), with representative correlation plots shown for assay plates 1 through 6. **d**, ATP, LYZ.NS, LYZ.S assay quality control comparisons across all plates and replicates, functional control wells chosen for comparison based on presence (S) or absence (NS) of Cch-stimulation in first (LYZ.NS) and second (LYS.S) secretion assays. One-tailed Welch’s t test for ATP; **** adj. p < 0.0001, one-way ANOVA post-hoc Dunnett’s multiple comparison test * adj. p = 0.0224, **** adj. p < 0.0001. **e**, Replicate strictly standardized mean difference (SSMD) for each assay in secondary validation screen, each point represents the SSMD from 8 well-replicates relative to DMSO control, orange signifies treatments passing cutoffs in both LYZ.NS and LYZ.S assays, red marking most potent compound, KPT-330. **f**, Flow cytometry gating strategy to select viable mature Paneth cells, final gate outlined in red. Source data

To screen for biological targets that may regulate Paneth cell differentiation in vitro and translate in vivo, we developed a scalable approach (thousands of samples) to scan a target-annotated small-molecule library and measure specific changes of a single cell type (Paneth cells) within a dynamic (differentiating) and heterogeneous (organoid) system, which represents the physiological differentiation environment (Fig. 1a). To model physiologically driven Paneth cell differentiation, we employed small-molecule-mediated enrichment and differentiation of murine adult-derived small intestinal organoids from ISCs (media formulated as ENR + CV – EGF, Noggin, R-spondin 1, CHIR99021, Valproic Acid – Methods) to Paneth cells (media formulated as ENR + CD – EGF, Noggin, R-spondin 1, CHIR99021, DAPT – Methods), as we have previously shown^10,11^. To scale this model, we adapted conventional 3D organoid culture into a 2.5D pseudo-monolayer, where ISC-enriched organoids are partially embedded on the surface of a thick layer of Matrigel at the Matrigel–media interface, rather than fully encapsulated in the Matrigel structure—an approach similar to others previously reported^29,30^. This technique enables Matrigel plating, cell seeding and media additions to be performed in a high-throughput, fully-automated, 384-well plate format and allows for analyte secretion directly into cell culture media (Extended Data Fig. 1a). To measure changes in Paneth cell abundance or quality, we used a validated assay measuring lysozyme (LYZ, an antimicrobial secreted specifically by Paneth cells) activity in cell culture media via a commercially available fluorescent reporter reaction that is readily implemented via automated liquid handling (Methods)^11^. Specifically, for each well, we first measured basally secreted LYZ (LYZ.NS), then carbachol (CCh)-induced secretion (LYZ.S) and finally, cellular adenosine triphosphate (ATP) as a measure of relative cell number per well. We assayed both stimulant-induced (LYZ.S – total cellular LYZ) and basal (LYZ.NS – constitutively secreted LYZ) secretion to distinguish compounds that may mediate changes in Paneth cell quality or secretion (LYZ.NS and LYZ.S uncorrelated) versus changes in Paneth cell abundance (LYZ.NS and LYZ.S correlated) (Extended Data Fig. 1b).

Using this pipeline, we performed a primary screen with a target-selective inhibitor library of 433 annotated compounds with high specificity to 184 unique biological targets (Supplementary Dataset 1) over a 6 d differentiation starting from ISC-enriched organoid precursors (*n* = 3 biological replicates originating from unique murine donors). In total, our proof-of-concept screen assayed 5,760 unique samples with the triplexed functional assay. Small molecules were added into distinct wells at 4 concentrations per compound (80 nm to 10 μM range) at day 0 and day 3, and on day 6, we measured basal and induced secretion of LYZ in media supernatants, as a specific marker of Paneth cell enrichment, as well as ATP. To verify multiplexed assay performance in the screen, randomly distributed dimethyl sulfoxide (DMSO)-treated wells and no-cell wells were placed in each screening plate, and DMSO-treated wells were exposed to CCh (stimulated) in varying orders across the two LYZ assays (Fig. 1a).

Following normalization of all measured wells (Methods), each assay had an approximate-normal distribution, with lower-value tails corresponding to toxic compounds (Extended Data Fig. 1b). Treatments across biological replicates and assays were well correlated, with Pearson correlation values between screen plates ranging from 0.50 to 0.74 (Extended Data Fig. 1c). Randomly placed control (DMSO) wells (labeled A,B,C,D based on stimulation order) had significantly higher ATP readings than no-cell wells (*P* < 0.0001), and in the LYZ.NS and subsequent LYZ.S assays, supernatant LYZ was significantly higher in 10 µM CCh-stimulated control wells than in basal control wells (A + B vs C + D LYZ.NS *P* < 0.0001, A vs B LYZ.S *P* < 0.05), which in turn were significantly higher than no-cell wells (no-cell vs A + B LYZ.NS *P* < 0.0001, no-cell vs A LYZ.S *P* < 0.0001) (Extended Data Fig. 1d). Small but statistically significant differences in control well assays across the entire screen are probably due to intrinsic variability in phenotypic screening in an organoid system, where precise control over cell number is limited, which in turn may impact non-normalized LYZ secretion measures. Accordingly, we chose to employ a strictly standardized mean difference (SSMD) methodology comparing replicate treatments to whole-plate controls so we could explicitly base our hit decision criteria within each assay on power calculations.

We next sought to define which molecules meaningfully increased Paneth cell abundance. We defined primary screen ‘hits’ as having replicate SSMDs greater than the calculated optimal critical value ($\beta _{\alpha _1}$ = 0.997) in both LYZ.NS and LYZ.S assays (Fig. 1b and Methods). This was determined as the intersection minimizing false positive and false negative levels (FPL and FNL error = 0.084) for upregulation of SSMD-based decisions^31^. The 47 hits correspond to treatment–dose (grouped by biological replicate) combinations that had a significant increase in LYZ.NS and LYZ.S without regard to viability (note that most hits based on these criteria had positive effects on cellular ATP). Hits were refined further to 15 treatment–dose combinations with the greatest biological effect, determined by a fold change in the top 10% of values for both LYZ assays relative to the plate (*z*-score > 1.282). Thus, 15 drugs (covering 18 treatment–dose conditions) from 13 unique annotated targets were identified as primary screen hits (Fig. 1c). For annotated targets with more than one hit, the most potent treatment–dose was selected for further investigation.

To validate primary screen hits against an ENR + CD (not plate) control, while refining dose-response ranges and narrowing hits to only the most potent activators of increased LYZ secretion, we performed a secondary screen with the 13 primary screen hit compounds. Compounds were tested at a narrowed dose range around each treatment’s identified optimal dose from the primary screen (4× below, 2× below and above). Hits in the validation screen were chosen as SSMDs greater than the calculated optimal critical value (*β*_*α*__1_ = 0.889) in both LYZ.NS and LYZ.S assays, with 6 compounds passing this threshold (Extended Data Fig. 1e). The same treatment–dose conditions passing the SSMD threshold also had the greatest biological effect, and in particular one compound, KPT-330, a known Exportin 1 (XPO1) inhibitor (a nuclear exporter that regulates the efflux of nuclear export signal (NES)-tagged cargoes, including many transcription factors, from the nucleus^32^), had two doses representing the greatest and near-greatest biological effect (~50–75% increases in LYZ.NS and LYZ.S relative to ENR + CD control) (Fig. 1d).

The results of primary and validation screening reflect a mixture of potential effects that may cause increases in total LYZ secretion. This includes contributions from: enhanced Paneth cell differentiation, altered Paneth cell quality and changes in total cell number concurrent with differentiation. To better inform how the 6 hit compounds increased total secreted LYZ, and to isolate only those that enhance Paneth differentiation robustly, we utilized flow cytometry to measure changes in Paneth cell representation within treated organoids. Concurrently, to ensure that we do not select compounds that manifest their behaviour only in specific in vitro settings, we performed the analyses in the conventional 3D culture method, controlling for 2.5D culture system-specific effects. Live Paneth cells were identified as LYZ-high, CD24-mid, side scatter-high (SSC-high) (Extended Data Fig. 1f). Only KPT-330—the most potent compound in validation screening—significantly enhanced the mature Paneth cell population within differentiating organoids, suggesting that KPT-330 induces Paneth differentiation (Fig. 1e). Of the 5 remaining compounds, Nilotinib excluded, none changed organoid composition and are probably driving changes in Paneth quality or are mediating effects dependent on 2.5D culture. Conversely, Nilotinib significantly decreased Paneth abundance while significantly increasing total cell number, suggesting that the overall increase in bulk LYZ secretion is an effect of increased proliferation, or 2.5D-mediated effect.

**Extended Data Fig. 2:**
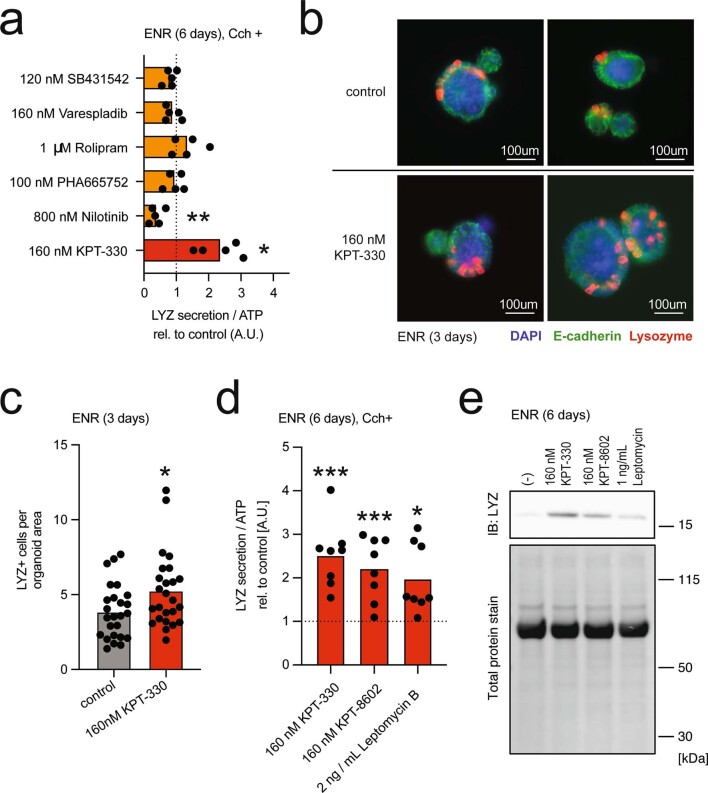
Profiling small molecule hits from Paneth cell-directed murine organoid screen. **a**, LYZ secretion assay for organoids differentiated in ENR with 6 hit compounds for 6 days. Organoids incubated in fresh basal media with 10 μM carbachol (Cch) for 3 h on day 6. All data normalized to ATP abundance and standardized to the control in each experiment. Means and individual values are shown (N = 5), dotted line represents the control value (1). One sample t-test compared to 1; **p = 0.0023, *p = 0.0101. **b**, Representative immunofluorescence images of 25 total organoids differentiated in ENR media with 160 nM KPT-330 for 3 days, Paneth cells marked by LYZ. **c**, Quantification of immunofluorescent images for LYZ + cells per organoid area for organoids differentiated in ENR with 160 nM KPT-330 for 3 days. Means and individual values are shown (N = 25 organoids). Unpaired two-tailed t-test; *p = 0.0275. **d**, LYZ secretion assay for organoids differentiated in ENR with 160 nM KPT-330, 160 nM KPT-8602 or 2 ng/mL Leptomycin B for 6 days. Organoids incubated in fresh basal media with 10 μM carbachol (Cch) for 3 h on day 6. All data normalized to ATP abundance and standardized to the control in each experiment. Means and individual values are shown (N = 5), dotted line represents the control value (1). One sample t-test compared to 1; ordered left to right, ***p = 0.0007, ***p = 0.0021, *p = 0.0095. **e**, Western blotting of intracellular LYZ in 3D-cultured intestinal organoids, cultured in ENR media for 6 days (N = 1). Source data

To examine whether our hits are dependent or independent of canonical stem cell niche signalling, we measured Paneth cell abundance in the ENR culture condition (removing CHIR99021 and DAPT, which mimic physiological Paneth differentiation through Wnt activation and Notch inhibition) in 3D (note that because Paneth cells exist in an immature state within ENR, we were unable to robustly quantify Paneth cell number via flow cytometry, and instead used the LYZ secretion assay). The result mirrored our flow cytometry findings in the ENR + CD condition, suggesting that the identified compounds act independently of strong Wnt and Notch drivers, and that only KPT-330 is enhancing Paneth cell-specific activity in the conventional organoid culture condition (Extended Data Fig. 2a). Collectively, these results led us to focus solely on understanding the activity mechanism of KPT-330.

### Support for XPO1 as a molecular target for enhancing Paneth cell differentiation

**Fig. 2:**
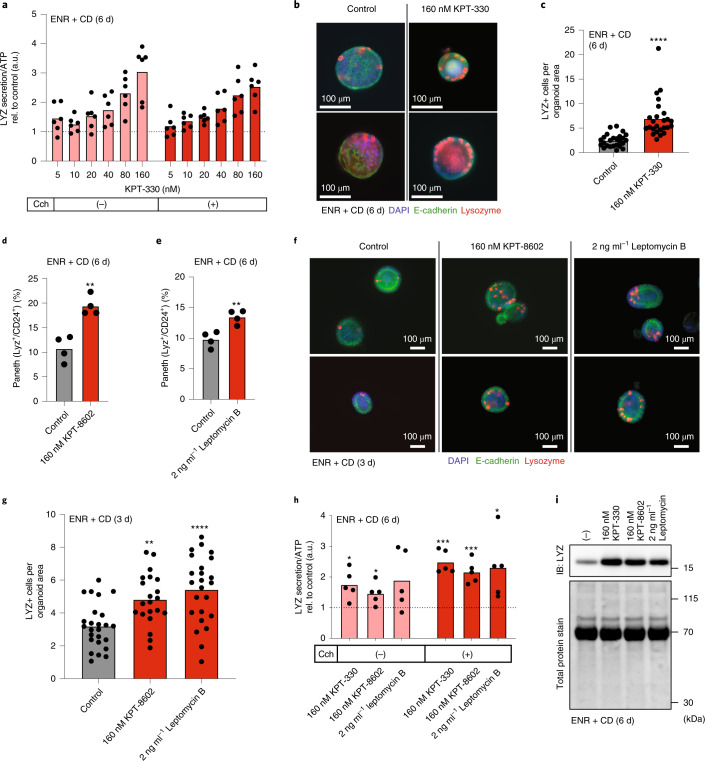
Small-molecule inhibition of XPO1 enhances Paneth cell differentiation. **a**, LYZ secretion assay for organoids differentiated in ENR+CD with increasing concentrations of KPT-330 for 6 d. Organoids were incubated in fresh basal media with or without 10 μM Cch for 3 h on day 6. All data are normalized to ATP abundance and standardized to the control in each experiment. Means and individual values are shown (*N* = 6), dotted line represents the control value (1). **b**, Representative immunofluorescence images of 25 total organoids differentiated in ENR+CD media with 160 nM KPT-330 for 6 d, Paneth cells are lysozyme (LYZ) positive. **c**, Quantification of immunofluorescence images for LYZ+ cells per organoid area for organoids differentiated in ENR+CD with 160 nM KPT-330 for 6 d. Means and individual values are shown (*N* = 25 organoids). Unpaired two-tailed *t*-test: ****P < 0.0001. **d**,**e**, Flow cytometry for Paneth cell fraction (lysozyme-positive and CD24-mid cells) of all live cells in 3D-cultured intestinal organoids treated with 160 nM KPT-8602 (**d**) and 2 ng ml^−1^ Leptomycin B (**e**) over 6 d culture in ENR+CD media. Means and individual values are shown (*N* = 4). Unpaired two-tailed *t*-test: (**d**) **P = 0.0019, (**e**) **P = 0.0051. **f**, Representative immunofluorescence images of 25 total organoids differentiated in ENR+CD media with 160 nM KPT-8602 or 2 ng ml^−1^ Leptomycin B for 3 d, Paneth cells are LYZ positive. **g**, Quantification of immunofluorescence images for LYZ+ cells per organoid area for organoids differentiated in ENR+CD with 160 nM KPT-8602 or 2 ng ml^−1^ Leptomycin B for 3 d. Means and individual values are shown (*N* = 25 organoids). One-way ANOVA post-hoc Dunnett’s multiple comparisons test: ****P = 0.0001, **P = 0.0035. **h**, LYZ secretion assay for organoids differentiated in ENR+CD with 160 nM KPT-330, 160 nM KPT-8602 or 2 ng ml^−1^ Leptomycin B for 6 d. Organoids were incubated in fresh basal media with or without 10 μM Cch for 3 h on day 6. All data were normalized to ATP abundance and standardized to the control in each experiment. Means and individual values are shown (*N* = 5), dotted line represents the control value (1). One-sample *t*-test compared to 1, ordered left to right: *P = 0.0242, *P = 0.0431, ***P = 0.0009, ***P = 0.0023. **i**, Western blotting of intracellular LYZ (immunoblot - IB) in 3D-cultured intestinal organoids cultured in ENR+CD media in the presence of KPT-330, KPT-8602 or Leptomycin B for 6 d (*N* = 1). Source data

We next sought to support the predicted on-target activity of KPT-330 and investigate the dose-dependency of treatment in enhancing Paneth cell differentiation. Administration of KPT-330 below 160 nM for 6 d (note that higher concentrations proved toxic in primary screening) showed LYZ secretion increasing in a dose-dependent manner, with 160 nM of KPT-330 being the most effective dose among tested concentrations (Fig. 2a). Immunofluorescent (IF) imaging for LYZ + Paneth cells within organoid cultures in ENR + CD + KPT-330 demonstrated an increase in Paneth cell number, in agreement with our results by both flow cytometry and LYZ secretion assay (Fig. 2b,c). Furthermore, this pro-Paneth differentiation effect was also observed by IF imaging in the ENR media +/− KPT-330 (Extended Data Fig. 2b,c). To demonstrate that XPO1 is the primary biological target of KPT-330, we used two additional XPO1 inhibitors: KPT-8602 and Leptomycin B^33^. Flow cytometry and IF imaging results show both KPT-8602 and Leptomycin B increasing the proportion of Paneth cells in the organoids (Fig. 2d–g). LYZ secretion assays with the additional XPO1 inhibitors show similar Paneth cell enrichments in both conventional (ENR) and Paneth-differentiating (ENR + CD) culture conditions (Fig. 2h and Extended Data Fig. 2d). Western blotting for intercellular LYZ per unit weight also confirms enrichment with each of the known XPO1 inhibitors in both conventional (ENR) and Paneth-differentiating (ENR + CD) culture conditions (Fig. 2i and Extended Data Fig. 2e), consistent with LYZ secretion, IF imaging and flow cytometry analyses.

KPT-330 (and KPT-8602) is a selective inhibitor of nuclear export (SINE); these molecules act by suppressing the XPO1-regulated nuclear export of multiple proteins and mRNAs from the nucleus to the cytoplasm—including genes involved in stem cell maintenance and differentiation as well as inflammatory stress response^34^. Additionally, XPO1 is known to regulate the cell cycle through its export-independent role in the regulation of mitosis^35^. On the basis of this evidence, we hypothesized that XPO1 inhibition via KPT-330 might provide for enhanced Paneth cell differentiation by directing ISCs to modulate their differentiation trajectories through alterations in either developmental signalling within the nucleus and/or interfering with the cell cycle.

### Single-cell RNA-sequencing of KPT-330-mediated differentiation reveals population shifts

**Fig. 3:**
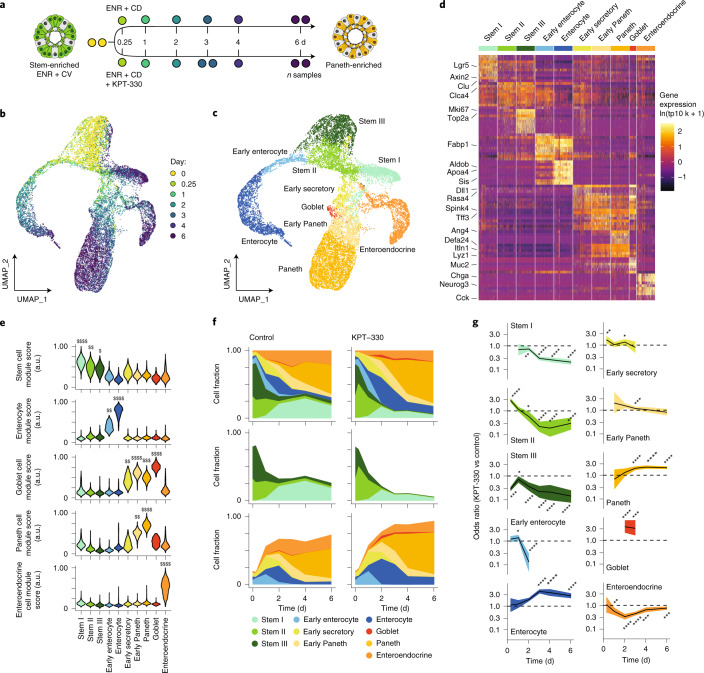
Longitudinal scRNA-seq profiling of organoid differentiation with KPT-330-mediated XPO1 inhibition. **a**, Stem-enriched (ENR+CV) to Paneth-enriched (ENR+CD) organoid differentiation in the presence and absence of 160 nM KPT-330; each circle represents a sample of organoids collected for scRNA-seq over the 6 d time course. **b**,**c**, UMAP of all samples labelled by differentiation timepoint (**b**) and annotated cell type (**c**). **d**, Log-normalized gene expression heat map for top 10 marker genes by cell type (by log fold change vs all others). **e**, Violin plots for all cell types representing module scores derived from gene sets enriched in in vivo intestinal stem cells, enterocytes, goblet cells, Paneth cells and enteroendocrine cells, with each score scaled on a range from 0 to 1. Effect size measured as Cohen’s *d*: $0.5 < *d* < 0.8, $$0.8 < *d* < 1.2, $$$1.2 < *d* < 2, $$$$*d* > 2. **f**, Organoid composition over time between untreated control and 160 nM KPT-330 treatment, for all cell types (top), stem cells (middle) and differentiating cells (bottom). **g**, Odds ratio enrichment and depletion over differentiation course based on Fisher exact testing, with 95% confidence interval plotted for each cell type relative to all others, dashed line at 1. FDR-adjusted Fisher exact testing: *P < 0.05, **P < 0.01, ***P < 0.001, ****P < 0.0001, individual *P* values reported in source data. Source data

**Extended Data Fig. 3:**
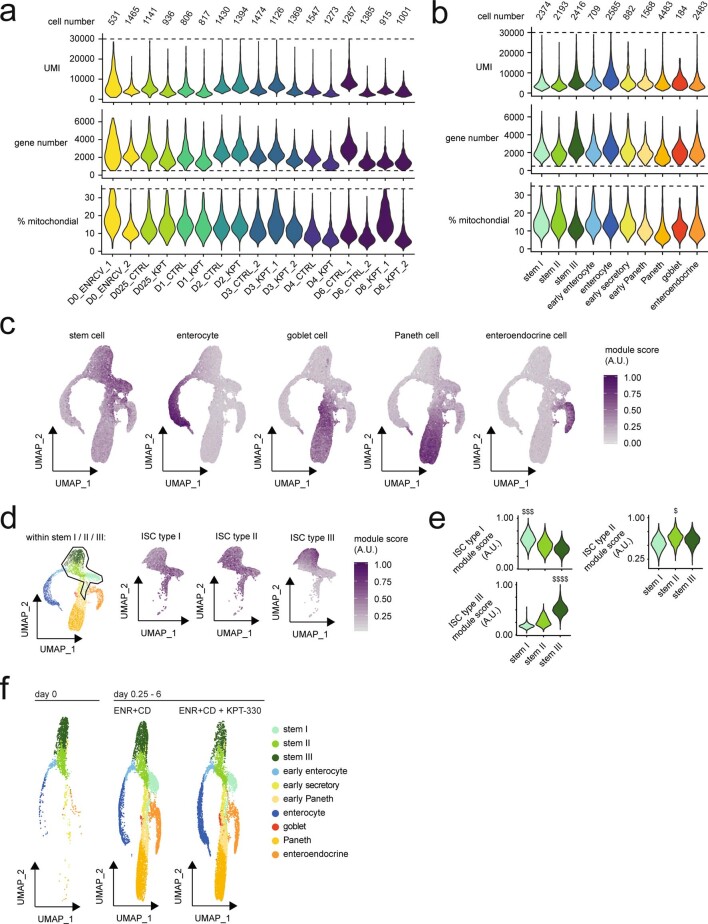
Single-cell RNA-seq profiling of murine organoid differentiation with KPT-330-mediated XPO1 inhibition. **a**, Single-cell RNA-seq quality metrics on a per-sample basis, including final cell number (barcodes) per array, and distributions of unique molecular identifiers per barcode (UMI), unique gene number per barcode, and percent of total UMIs corresponding to mitochondrial genes per barcode. **b**, Single-cell RNA-seq quality metrics on a per-cell type basis, including final cell number (barcodes) per array, and distributions of unique molecular identifiers per barcode (UMI), unique gene number per barcode, and percent of total UMIs corresponding to mitochondrial genes per barcode. **c**, Feature plots over organoid differentiation UMAP representing module scores derived from gene sets enriched in in vivo stem cells, enterocytes, goblet cells, Paneth cells, and enteroendocrine cells, each score scaled on a range from 0 to 1. **d**, Feature plots over organoid differentiation UMAP restricted to stem I / II / III populations representing module scores derived from gene sets enriched in *in vivo* type I / II / III intestinal stem cells (ISCs), each score scaled on a range from 0 to 1. **e**, Violin plots for stem I / II / III populations representing module scores derived from gene sets enriched in *in vivo* type I / II / III intestinal stem cells (ISCs), each score scaled on a range from 0 to 1. Effect size measured as Cohen’s d, $ 0.5 < d < 0.8, $$$ 1.2 < d < 2, $$$$ d > 2. **f**, Organoid differentiation UMAP labeled by annotated cell type, and split by day 0 (ENR + CV), day 0.25–6 control (ENR + CD), and day 0.25–6 160 nM KPT-330-treated (ENR + CD + KPT-330). Source data

To test the hypothesis that KPT-330 drives Paneth differentiation by altering ISC behaviour, we utilized single-cell RNA-sequencing (scRNA-seq) via Seq-Well S^3^ (ref. ^36^). We performed a longitudinal comparison between untreated and KPT-330-treated organoids over a 6 d differentiation, with particular emphasis on early timepoints (Fig. 3a). We collected 17 samples at the following timepoints: 6 h (0.25 d) and 1, 2, 3, 4 or 6 d. Each sample consists of single cells from >1,000 organoids from pre-differentiation ENR + CV organoids and both ENR + CD and ENR + CD + KPT-330 (160 nM) conditions. For timepoints beyond 2 d, media were refreshed every other day. The resulting dataset consists of 19,877 cells. Unique molecular identifier (UMI), percent mitochondrial and detected gene distributions are similar across samples, within acceptable quality bounds (genes >500, UMI <30,000, percent mitochondrial <35) (Extended Data Fig. 3a).

Following normalization, variable feature selection and principal component dimensional reduction (Methods), uniform manifold approximation and projection (UMAP) visualization of the complete dataset reveals the time-course structure, along with branches suggestive of distinct lineages arising over the course of differentiation (Fig. 3b). Tiered Louvain clustering separated the data into 10 clusters, which we manually annotated (Fig. 3c) on the basis of marker gene expression corresponding to canonical markers of intestinal epithelial cell types (Fig. 3d and Supplementary Dataset 2). Each cluster possessed similar quality metrics, suggesting that clusters are driven by biological and not technical differences (Extended Data Fig. 3b). To contextualize and provide a more robust measure of cellular identity of our 10 clusters, we used lineage-defining gene sets from a murine small intestinal scRNA-seq atlas^37^ to score for enrichment in gene-set expression (Extended Data Fig. 3c). The 10 clusters include 3 stem-like, 2 enterocyte, 1 early secretory, 1 goblet, 2 Paneth and 1 enteroendocrine, aligning with our expectation that ENR + CD differentiation should enrich for secretory epithelial cells—principally Paneth and to a lesser extent goblet and enteroendocrine (Fig. 3e). To distinguish the 3 stem-like clusters and assess physiological relevance, we performed module scoring over gene sets identified to correspond to known ISC subsets in vivo^6^ (Extended Data Fig. 3e). We see alignment with the type III and type I ISCs, along with slight enrichment for a distinct type II (Extended Data Fig. 3e), although this population may also be an intermediate between stem I and III populations, sharing markers with both (Fig. 3d). Accordingly, we adopted this naming scheme to describe the 3 ISC populations: type I, enriched for canonical markers of ISCs (including LGR5); type III, distinguished by the high expression of cell cycle genes; and type II, appearing as a transitory or intermediate population between I and III.

We next explored changes in cell type representation between organoids treated with KPT-330 versus the control. In the combined dataset, we do not observe cell clusters unique to the KPT-330 treatment, but rather shifts in cluster composition (Extended Data Fig. 3f). Both conditions begin with over 75% of cells being either stem II or stem III. By day 2, stem I emerges, accounting for approximately 25% of the cells in the control condition, but a smaller proportion in KPT-330-treated organoids. Early enterocytes emerge at day 1, with the continued differentiation to enterocytes peaking at day 2 and becoming less prevalent by day 4. Early secretory, goblet and early Paneth populations appear to crest with enterocytes, followed by a transition to Paneth cells continuing to day 6 (Fig. 3f). To better quantify the differences in representation between the KPT-330 and control conditions over time, we performed Fisher’s exact testing for each cell type relative to all others. This was done for each timepoint when that cell type accounted for at least 0.5% of cells in both KPT-330 and control samples. We present the relative enrichment or depletion of a cell population with KPT-330 treatment over time as the odds ratio with a corresponding 95% confidence interval. KPT-330 treatment leads to a depletion of stem I, II, III and enteroendocrine cells over time, along with the corresponding enrichment of enterocytes, goblet (NB in this system goblet cells represent a very small fraction of total cells) and Paneth cells (Fig. 3g). The observed twofold enrichment in Paneth cells at day 6 mirrors our flow cytometry observations of a twofold increase in mature Paneth cells, while also showing the unexpected early enrichment of enterocytes and longer-term depletion of a subset of stem cells—the quiescent stem I population.

### KPT-330 alters signalling and transcription factor activity across organoid cell types

**Fig. 4:**
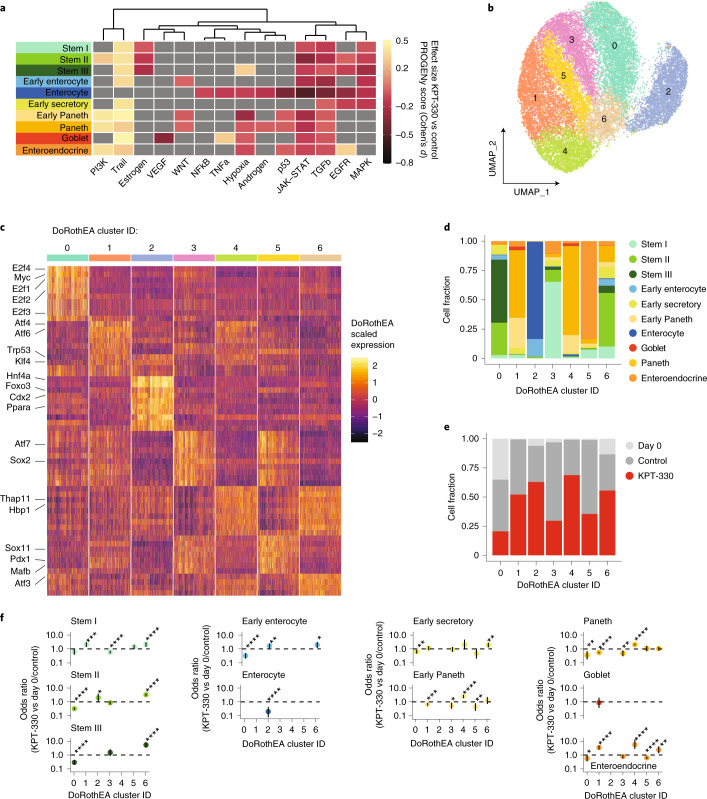
Inferred signalling pathway activity and upstream transcription factors associated with KPT-330-mediated differentiation. **a**, Heat map of Cohen’s *d* effect sizes (greyed values <±0.2) comparing differential PROGENy pathway activities between KPT-330 and non-treated cells within each cell type. **b**, Organoid scRNA-seq UMAP based on upstream TF prediction (DoRothEA) of all samples labelled by TF clustering (clusters named 0-6). **c**, Scaled predicted upstream transcription factor activity heat map for top 10 markers by TF cluster (by log fold change vs all others). **d**,**e**, Stacked bar chart for DoRothEA cluster by cell type (**d**) and KPT-treatment (**e**). **f**, Odds ratio enrichment and depletion by cell type and over DoRothEA cluster based on Fisher exact testing, with 95% confidence interval plotted for each cell type relative to all others, dashed line at 1. FDR-adjusted Fisher exact testing: *P < 0.05, **P < 0.01, ***P < 0.001, ****P < 0.0001, individual *P* values reported in source data. Source data

**Extended Data Fig. 4:**
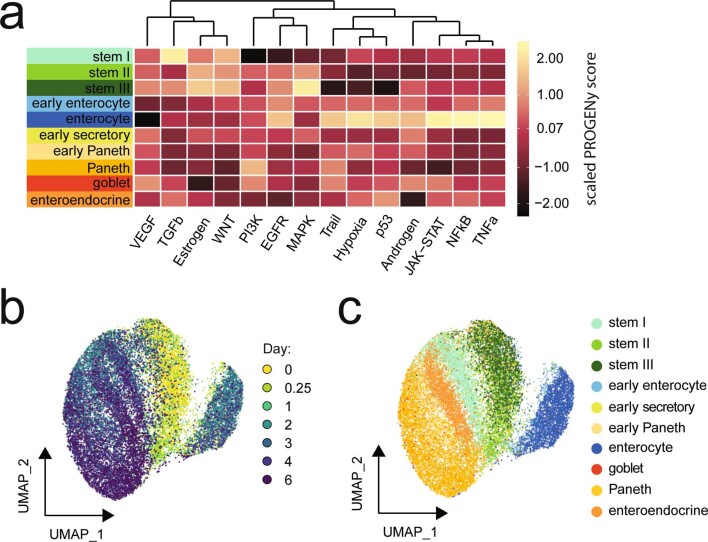
Pathway and inferred transcription factor landscape of single-cell RNA-seq data from murine organoid differentiation. **a**, Heatmap of predicted pathway signaling for non-KPT-330-treated cells using PROGENy scores calculated over single-cell RNA-seq data for each cell type. **b**, Organoid single-cell RNA-seq UMAP based on upstream transcription factor (TF) prediction (DoRothEA) of all samples labeled by differentiation timepoint. **c**, Organoid single-cell RNA-seq UMAP based on upstream transcription factor (TF) prediction (DoRothEA) of all samples labeled by annotated cell type. Source data

To clarify the mechanism, determine potential mediators of putative KPT-330-mediated XPO1 inhibition in our system and better understand the differentiation process, we performed signalling pathway and upstream transcription factor (TF) inference on our scRNA-seq dataset using the PROGENy (to infer signalling pathway activity) and DoRothEA (to infer upstream TF activity) toolsets^38^ (Methods). First, we examined how signalling pathway activity is distributed across the untreated cells of our differentiation system (Extended Data Fig. 4a), revealing expected pathways such as Wnt enriched across stem populations, and nuclear factor kappa-light-chain-enhancer of activated B cells (NFkB) and tumor necrosis factor alpha (TNF-alpha) enriched in enterocytes, suggesting that PROGENy captures meaningful biology. Within stem III, we observe high levels of mitogen-activated protein kinase (MAPK) and epidermal growth factor receptor (EGFR) signalling and low levels of stress-associated Trail and Hypoxia signalling. We next sought to understand how KPT-330 treatment effects signalling by computing the effect size (Cohen’s *d*) of KPT-330 treatment for each pathway and cell type (Fig. 4a). We observe a pan-epithelial increase in stress-associated Trail signalling, stem III-specific increase in stress-associated hypoxia signalling, and secretory cell-specific increase in phosphatidylinositol 3-kinase (PI3K) signalling. In addition, Janus kinase - signal transducer and activator of transcription protein (JAK-STAT) and transforming growth factor-beta (TGF-beta) are decreased across epithelial cell types, while EGFR and MAPK are decreased within the stem and early differentiating populations. These observations suggest decreases in mitogen signalling restricted to stem and progenitor populations, along with broader increases in cell stress responses. To determine potential transcriptional regulators associated with these changes, we performed UMAP visualization and Louvain clustering over inferred TF activity of the full single cell dataset, resulting in 7 clusters corresponding to upstream TF states (Fig. 4b). This upstream TF landscape captures heterogeneity associated with differentiation time and cell type (Extended Data Fig. 4b,c). The 7 states are distinguished by enrichment for cell cycle-associated TFs (0), known TFs of cell type-specific differentiation (2, enterocytes, Hnf4; and 5, enteroendocrine, Pdx1), TFs representing distinct stress responses (1, Atf4/6; and 6, Atf3, Thap11) and intermediary states of the aforementioned (3 and 4) (Supplementary Dataset 3 and Fig. 4c). Multiple clusters are enriched for distinct cell types associated with their cellular programmes (0, cycling stem; 2, enterocytes; 5, enteroendocrine), while others suggest programmes differentially induced by KPT-330 treatment (Paneth-enriched 1 and 4, and progenitor-enriched 6) (Fig. 4d,e). To quantify the differences in representation between KPT-330 and control conditions in each of the 7 transcriptional states, we performed Fisher’s exact testing for each cell type relative to all others. This was done for each cluster where that cell type accounted for at least 10 cells in both KPT-330 and control samples. KPT-330 treatment leads to a depletion of cell cycle cluster 0 and enrichment in stress response cluster 6 across stems I, II, III, early enterocyte, early secretory and enteroendocrine cells, along with shifts in Paneth cells from clusters 1 to 4 (Fig. 4f). These analyses point to stem and progenitor-specific changes in the cell cycle and broader induction of stress responses following KPT-330-mediated XPO1 inhibition over the course of differentiation.

### KPT-330 induces ISC differentiation via stress response and suppressed mitogen signalling

**Fig. 5:**
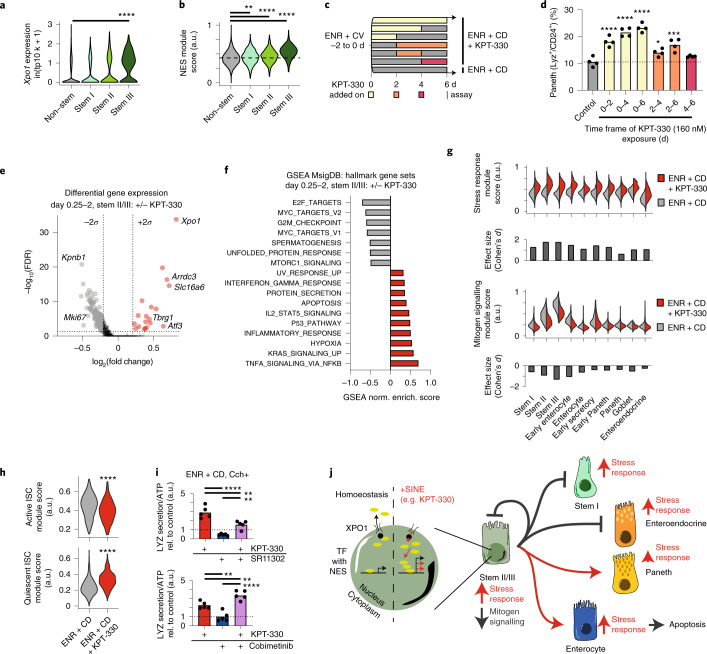
KPT-330-mediated XPO1 inhibition drives stem cell-specific and pan-epithelial responses to induce differentiation. **a**, Violin plots of scRNA-seq log-normalized (transcripts per 10,000 – tp10k) expression of *Xpo1* in all untreated control cells split by non-stem and stem I/II/III annotations. Wilcoxon rank sum test, Bonferroni correction stem I/II/III vs non-stem: ****P < 0.0001. **b**, Violin plots of module scores over all cells derived from genes with known NES in all untreated control cells split by non-stem and stem I/II/III annotations, with each score scaled on a range from 0 to 1. One-way ANOVA post-hoc Dunnett’s multiple comparisons test, ordered left to right: **P < 0.0063, ****P < 0.0001. **c**, Time course of KPT-330 treatment of ENR+CD differentiating organoids, with treatments over every continuous 2, 4 and 6 d interval. **d**, Flow cytometry analyses of 3D-cultured intestinal organoids treated with KPT-330 for the indicated time frame during 6 d culture in ENR+CD media. Paneth cells were identified as lysozyme-positive and CD24-positive cells. Means and individual values are shown (*N* = 4), and the dotted line represents the average of the Paneth cell fractions in control samples. One-way ANOVA post-hoc Dunnett’s multiple comparisons test, ordered left to right: ****P < 0.0001, *P = 0.0323, ***P = 0.001. **e**, Volcano plot of differentially expressed scRNA-seq log-normalized genes between KPT-330-treated and control cells within stem II/III populations in early timepoints (day 0.25–2). Red points, enriched in KPT-330 treatment; grey, enriched in control. Differential expression based on Wilcoxon rank sum test with significant log_2_ fold changes based on ±2*σ* (dotted lines) of all genes; FDR (Bonferroni correction) cutoff *P* < 0.05. **f**, GSEA normalized enrichment score over all differentially expressed genes between KPT-330-treated and control cells within stem II/III populations in early timepoints (day 0.25–2). Gene sets shown from MSigDB Hallmark v7 with FDR < 0.05; red, enriched in KPT-330 treatment; grey, enriched in control. **g**, Violin plots split by KPT-330-treated and control for module scores derived from significantly enriched (stress response) and depleted (mitogen signalling) genes in KPT-330-treated and control cells within stem II/III populations in early timepoints (day 0.25–2); each score was scaled on a range from 0 to 1. Effect size (Cohen’s *d*) for each module between KPT-330-treated and control modules within each cell type is represented in bar chart below violin plots. **h**, Violin plots of module scores derived from genes expressed in active and quiescent ISCs between KPT-330-treated and control cells within stem II/III populations in early timepoints (day 0.25–2); each score was scaled on a range from 0 to 1. Two-sided *t*-test: ****P < 0.0001. **i**, LYZ secretion assay for organoids differentiated in ENR+CD, treated with 10 μM SR11302 (AP-1 inhibitor) or 20 nM Cobimetinib (MEK inhibitor) for 6 d. Organoids were incubated in fresh basal media with or without 10 μM Cch for 3 h on day 6. All data were normalized to ATP abundance and standardized to the control in each experiment. Means and individual values are shown (*N* = 5), dotted line represents the control value (1). One-way ANOVA post-hoc Tukey’s multiple comparisons test, ordered left to right, top to bottom: ****P < 0.0001, **P = 0.0014, **P = 0.0055, **P = 0.0035, **P = 0.0085, ****P < 0.0001. **j**, Proposed mechanism for XPO1 inhibition driving transcriptional changes manifesting as increased stress responses and reduced mitogen signalling, resulting in re-balanced cycling of stem cell fate decisions towards secretory Paneth cells and absorptive enterocytes. Source data

**Extended Data Fig. 5:**
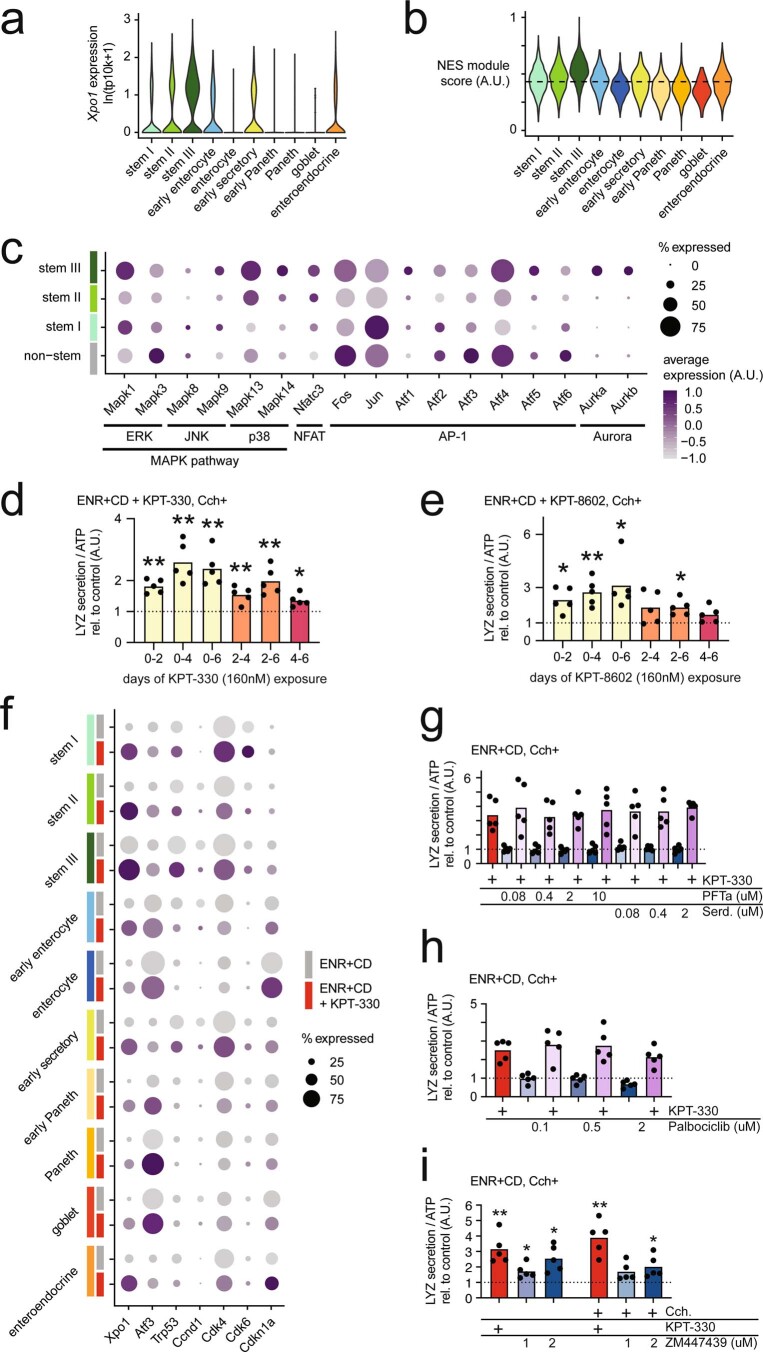
Signaling pathway analysis of murine organoids with KPT-330-mediated XPO1 inhibition. **a**, Single-cell RNA-seq log normalized (transcripts per 10,000 – tp10k) expression of *Xpo1* in control cells by cell type annotations. **b**, Violin plots of nuclear export signal (NES) containing gene module score in control cells by cell type annotations, scaled from 0 to 1. **c**, Single-cell RNA-seq log normalized expression of genes involved in MAPK, NFAT, AP-1, and Aurora kinase signaling in control cells by non-stem, and stem I / II / III annotations. **d**, LYZ secretion assay for organoids treated with KPT-330 (**d**) or KPT-8602 (**e**) for 6 days culture in ENR + CD media. Organoids were incubated in fresh basal media with 10 μM carbachol (Cch) for 3 h on day 6. Data normalized to ATP and standardized to the control in each experiment. Means and individual values are shown (N = 5), dotted line represents the control value (1). One sample two-tailed t-test compared to 1; significant p values for (**d**) ordered left to right, **p = 0.0011, **p = 0.0049, **p = 0.0053, **p = 0.0098, **p = 0.0077, *p = 0.0156, for (**e**) ordered left to right, *p = 0.0132, **p = 0.0071, *p = 0.0295, *p = 0.0124. **f**, Single-cell RNA-seq log normalized expression of genes regulated by XPO1 signaling between KPT-330-treated and control cells by cell-type annotations. Color scale is relative to control, purple-to-grey increasing relative-expression. LYZ secretion assay for organoids treated with p53 modulators, inhibitor Pifithrin-α (PFTa) and activator Serdemetan (Serd.) (**g)**, CDK4/6 inhibitor Palbociclib (**h**), Aurora kinase inhibitor ZM447439 (**i**), over 6-day culture in ENR + CD media with or without 160 nM KPT-330. Organoids were incubated in fresh basal media with 10 μM carbachol (Cch) for 3 h on day 6. Data normalized to ATP and standardized to control in each experiment. Means and individual values are shown (N = 5), and the dotted line represents the control value (1). One sample two-sided t-test compared to 1; significant p-values for (**i**) ordered left to right, **p = 0.0073, *p = 0.0259, *p = 0.0151, **p = 0.0040, *p = 0.0331. Source data

Compositional, signalling and upstream TF changes during differentiation with KPT-330 are consistent with XPO1 inhibition acting on stem II/III populations. In untreated organoids, the expression of *Xpo1* is significantly enriched in the cycling stem III population (Fig. 5a and Extended Data Fig. 5a), and the expression of genes known to contain a NES (which is required for the nuclear efflux via XPO1) is enriched in the stem cell populations—most significantly in stem III (Fig. 5b and Extended Data Fig. 5b)^39^. XPO1 is known to mediate nuclear signalling processes including the MAPK pathway, nuclear factor of activated T-cells (NFAT), activator protein-1 (AP-1) and Aurora kinase activity during cell division^32,34^. With this in mind, we observe the expression of many key mediators in these pathways within the stem populations (in agreement with our observations in Extended Data Fig. 4a), and see particular stem II and III expression in members of MAPK (*Mapk1*, *Mapk9, Mapk13, Mapk14*), NFAT (*Nfatc3*), AP-1 (*Fos, Jun, Atf1-6*) and Aurora kinases (*Aurka, Aurkb*) (Extended Data Fig. 5c).

To further validate whether the stem II/III population is the principal cellular target of KPT-330-mediated XPO1 inhibition, we leveraged the dynamic nature of our system and exposed organoids to KPT-330 over every 2, 4 and 6 d interval in the 6 d differentiation, and measured final abundance and function of mature Paneth cells at day 6, thereby inferring the relative effect of XPO1 inhibition on each cell type (Fig. 5c). Of all the 2 d KPT-330 treatments, day 0–2 results in the greatest enrichment in mature Paneth cells, with longer exposure after day 2 providing additional, albeit lesser enrichment. Furthermore, day 2–4 treatment produces moderate enrichment, while day 4–6 treatment is not different (by flow cytometry) or is slightly enriched (by LYZ secretion assay) from the untreated (Fig. 5d and Extended Data Fig. 5d). Using an additional SINE, KPT-8602, we observe similar enrichment behaviour as KPT-330 (Extended Data Fig. 5e). These data are consistent with XPO1 inhibition altering stem II/III differentiation – the largest effects of XPO1 inhibition are concurrent with periods in the differentiation course where stem II/III populations are most abundant. However, these data also suggest that XPO1 inhibition may not be entirely stem-dependent, given the lesser but significant increases in Paneth cell number and function with later treatment, where stem II/III populations are greatly diminished.

To better understand the pleiotropic effects of KPT-330-mediated XPO1 inhibition that may mediate differentiation, we examined the differentially expressed genes between KPT-330-treated and untreated stem II/III populations in the earliest stages of differentiation when they are most abundant (day 0.25–2). Both the most significantly enriched (*Xpo1*) and depleted (*Kpnb1*, a nuclear importin) genes suggest that these cells are significantly impacted by KPT-330 treatment and are enacting changes in expression to re-establish homoeostasis of nuclear cargo transit (Fig. 5e and Supplementary Dataset 4). Additional notable genes with significantly increased expression include *Arrdc3* (regulates proliferative processes), *Slc16a6* (principal transporter of ketone bodies; instructional in ISC fate decisions), *Tbgr1* (growth inhibitor) and *Atf3* (regulates stress response in ISCs)^40–43^. Genes downregulated by KPT-330 treatment appear related to proliferation and the cell cycle, including the marker *Mki67*. In addition to substantial changes within early stem II/III populations, genes regulated by XPO1 inhibition—including *Xpo1*, *Atf3*, *Trp53* (p53)*, Ccnd1, Cdk4/6* and *Cdkn1a* (p21)—have increased expression across all cell types (at all times), but with significant differences in the fraction of cells that express each gene (Extended Data Fig. 5f). This suggests that there are both stem II/III-specific responses and pan-epithelial responses to XPO1 inhibition.

To better contextualize the transcriptional response to KPT-330 treatment in stem II/III cells, we performed gene-set enrichment analyses (GSEA) using the v7 molecular signatures database (MSigDB) hallmark collection, which represent specific well-defined biological states or processes across systems^44,45^. Significant gene sets with FDR < 0.05 reveal two major programmes differentially enriched following KPT-330 treatment, with enrichment or depletion quantified through the GSEA normalized enrichment score (Fig. 5f and Supplementary Dataset 4). KPT-330 treatment suppresses programmes downstream of mitogen-driven signalling—notably, targets of E2F and MYC, as well as genes involved in the cell cycle (G2M checkpoint)—while upregulating programmes broadly resembling a stress response (NFkB signalling, hypoxia, inflammatory response), in agreement with our PROGENy and DoRothEA observations and with the known effects of XPO1 inhibition in the context of malignancy.

We next examined whether the responses embodied by the significant differentially expressed genes in stem II/III (day 0.25–2) may be pan-epithelial or restricted to the cycling stem II/III populations. The stress response module (differentially increased in stem II/III) is substantially increased across all cells during differentiation, with the greatest effect in the stem II/III as well as in early mature cell populations, and the lowest effect in the mature Paneth cells (Fig. 5g). Conversely, the mitogen signalling module (differentially decreased in stem II/III) is selectively decreased in stem II/III and early enterocyte populations relative to all others. This selectivity corresponds with our observation that the majority of mitogen signalling occurs within the proliferative stem II/III populations relative to the mature populations. As further evidence of altered mitogen signalling impacting stem II/III cells following XPO1 inhibition, we observe a decrease in a previously identified gene module^46^ of active ISCs, and a corresponding increase of the quiescent ISC module in our early (day 0.25–2) stem II/III cells (Fig. 5h). Combined with our observation that XPO1 inhibition blocks the emergence of the quiescent stem I population, our data suggest a model wherein SINE-induced stress response and disruption of mitogen signalling instruct proliferative progenitors to exit the cell cycle and differentiate preferentially towards the Paneth lineage, while limiting the accumulation of quiescent stem I cells and enteroendocrine cells.

We sought to clarify this conceptual model with the use of additional small-molecule inhibitors known to modulate discrete components of our hypothesized differentiation process, namely: signalling through XPO1-associated stress response including AP-1 and p53, signalling within the MAPK pathway and finally, XPO1-mediated effects on mitosis through association with Aurora kinases. We began by treating organoids along the ENR + CD differentiation course with SR11302, a small-molecule inhibitor of AP-1, to test whether AP-1 is critical to the SINE-induced stress response, both alone and in combination with KPT-330. We observe that SR11302 significantly decreases functional LYZ secretion at the end of the 6 d differentiation, both in combination with KPT-330 and alone (Fig. 5i). This suggests that AP-1 signalling is a mediator of Paneth differentiation from ISCs.

We next tested whether p53 is a downstream mediator of XPO1 inhibition by repeating the above assay with two known p53 modulators: a p53 inhibitor pifithrin-α (PFTa) and p53 agonist serdemetan (serd.). Across a wide dose range, both p53 modulators tested did not alter Paneth cell differentiation—neither alone nor in combination with KPT-330—suggesting that the KPT-330 stress response is not dependent on p53 signalling modulated by either compound (Extended Data Fig. 5g). With the same assay, we probed the mitogen signalling response by adding the mitogen-activated protein kinase kinase (MEK) inhibitor, cobimetinib (previously shown to induce the quiescent ISC population^46^), in combination with KPT-330. Cobimetinib alone did not significantly alter Paneth cell differentiation, but it synergized with KPT-330 (Fig. 5i) to increase Paneth cell differentiation. We next sought to test whether the regulation of the cell cycle via mitogen signalling may act as a downstream mediator following XPO1 inhibition. Inhibition of Cdk4/6 with palbociclib both alone and in combination with KPT-330 did not alter Paneth cell differentiation (Extended Data Fig. 5h), but inhibition of Aurora kinase B with ZM447439 did significantly increase Paneth cell differentiation (notably, ZM447439 was also a lower-effect-size hit in our primary screen) (Extended Data Fig. 5i). Combined, these experiments suggest that the SINE-induced stress response may be mediated by AP-1 but not p53, while suppression of mitogen signalling is not dependent on extracellular signal-regulated kinases (ERK), but is further enhanced by ERK inhibition. Additionally, the non-exporter-related action of XPO1 during the cell cycle (which interacts with Aurora kinase) may further contribute to the observed pro-differentiation effect.

In total, our analyses suggest that KPT-330-mediated XPO1 inhibition drives Paneth cell enrichment through the modulation of cell state within cycling ISCs (stem II/III). Further, this modulation includes a confluence of pan-epithelial stress response and suppression of mitogen signalling within stem II/III. We observe the cycling stem population becoming transiently quiescent, thereby favouring differentiation towards the Paneth and enterocyte lineages (the latter being a short-lived population relative to the former) over a more balanced transition to the mature lineages and the quiescent stem pool (stem I) (Fig. 5j).

### KPT-330 mediates pro-barrier differentiation in human organoids

**Fig. 6:**
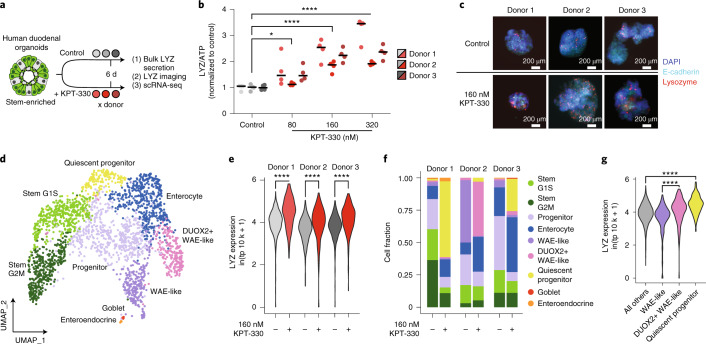
KPT-330-mediated XPO1 inhibition in human SI organoids has pro-differentiation effects that mirror those in the murine system. **a**, Stem-enriched human small intestinal organoid differentiation in the presence and absence of KPT-330. Each circle represents a sample of organoids collected from a unique donor for a LYZ secretion assay, IF imaging and scRNA-seq over the 6 d time course. **b**, LYZ secretion assay for human organoids treated with increasing concentrations of KPT-330 for 6 d. Organoids were incubated in fresh basal media with 10 μM Cch for 3 h on day 6. All data were normalized to ATP abundance and standardized to the control in each experiment. Donor means and individual values are shown (*N* = 4). One-way ANOVA post-hoc Dunnett’s multiple comparisons test, ordered left to right: *P = 0.0114, ****P < 0.0001. **c**, Representative immunofluorescence images of human organoids treated with 160 nM KPT-330 for 6 d; images from single experiment are shown. **d**, Organoid UMAP of all samples labelled by annotated cell type. **e**, Violin plot of scRNA-seq log-normalized (transcripts per 10,000 – tp10k) expression of *LYZ* in all cells, grouped by donor and split by KPT-330 treatment. Wilcoxon rank sum test, treated vs non-treated: ****P < 0.0001. **f**, Stacked bar chart for annotated cell type by cell type, grouped by donor and split by KPT-330 treatment. **g**, Violin plot of scRNA-seq log-normalized (transcripts per 10,000 – tp10k) expression of *LYZ* in all donors, grouped by cell type annotation. Wilcoxon rank sum test, DUOX2+ WAE-like vs WAE-like and quiescent progenitor vs all others: ****P < 0.0001. Source data

To extend our observations from the murine organoid system, we sought to examine how XPO1 inhibition via KPT-330 may affect human small intestinal stem cells by utilizing a stem-cell-enriched human small intestinal organoid model^47^ (Methods). This model (as is the case for other reported human small intestinal organoid models) does not generate an appreciable mature Paneth cell population, probably owing to an absence of critical niche signalling factors. Thus, we sought to assess whether KPT-330 would alter patterns of stem cell differentiation (Fig. 6a), in a fashion consistent with our murine models. We performed a dose-response study with KPT-330 in a human-derived stem cell-enriched small intestinal organoid model from three unique duodenal donors and found that normalized LYZ secretion significantly increases with dose (Fig. 6b). At a single dose (160 nM KPT-330, equivalent to our murine organoid scRNA-seq study), we conducted IF imaging for LYZ and observed increased LYZ+ cell abundance (Fig. 6c). In both LYZ secretion and imaging morphology, we see that there may be donor-dependent responses to KPT-330 treatment (note donor 1 appearing most responsive and donor 2 least).

**Extended Data Fig. 6:**
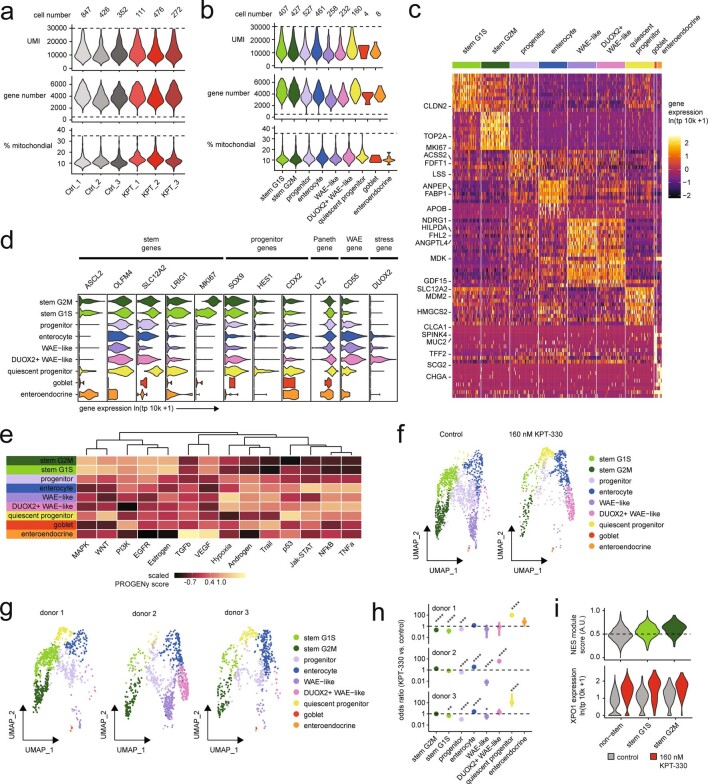
Single-cell RNA-seq analysis for human organoids. **a**, Human organoid single-cell RNA-seq quality metrics on a per-sample basis, including final cell number (barcodes) per array, and distributions of unique molecular identifiers per barcode (UMI), unique gene number per barcode, and percent of total UMIs corresponding to mitochondrial genes per barcode. **b**, Human organoid single-cell RNA-seq quality metrics on a per-cell type basis, including final cell number (barcodes) per array, and distributions of unique molecular identifiers per barcode (UMI), unique gene number per barcode, and percent of total UMIs corresponding to mitochondrial genes per barcode. **c**, Log normalized gene expression heatmap for top 10 marker genes by cell type (by log fold change vs. all others). **d**, Single-cell RNA-seq log normalized expression of known marker genes for stem, progenitor, Paneth, wound associated epithelium (WAE), and stress response, grouped by cell-type annotations. **e**, Heatmap of predicted pathway signaling for all human organoid cells using PROGENy scores calculated over single-cell RNA-seq data for each cell type. **f**, Human organoid UMAP labeled by annotated cell type, and split by treatment (control and KPT-330). **g**, Human organoid UMAP labeled by annotated cell type, and split by donor. **h**, Odds ratio enrichment and depletion by cell type and over donor based on Fisher exact testing with 95% confidence interval for each cell type relative to all others, dotted line at 1. FDR-adjusted Fisher exact testing; *p < 0.05, **p < 0.01, ***p < 0.001, ****p < 0.0001, individual p-values reported in source data. **i**, Violin plots of module scores over all cells derived from genes with known nuclear export signals (NES) in all un-treated control cells split by non-stem, and stem G1S / G2M annotations, each score scaled on a range from 0 to 1 with below violin plot of *XPO1* expression grouped by stem classification and split by treatment. Source data

To more completely explore the changes induced by KPT-330 in this human organoid model, and any donor associated effects, we again utilized scRNA-seq via Seq-Well S^3^. We performed a comparison between untreated and KPT-330-treated organoids from each donor over a 6 d differentiation, with each sample consisting of single cells from >1,000 organoids. The resulting dataset consists of 2,484 cells. UMI, percent mitochondrial and detected gene distributions are similar across samples, within acceptable quality bounds (genes >500, UMI <30,000, percent mitochondrial <35) (Extended Data Fig. 6a).

Following normalization, variable feature selection and principal component analysis (Methods), tiered Louvain clustering separated the data into 7 clusters with similar quality metrics (Extended Data Fig. 6b), which we manually annotated (Fig. 6d) on the basis of the expression of canonical marker genes of intestinal epithelial cell types (Extended Data Fig. 6d,e). To further determine the cellular identity of our 7 clusters, we used PROGENy to infer signalling pathway activity in each population (Extended Data Fig. 6e). The 7 clusters include 2 stem-like clusters (differing stages of cell cycle), 2 progenitor subsets, 2 clusters with features consistent with wound-associated epithelium (WAE-like), and a joint enterocyte, goblet and enteroendocrine cluster. The 2 WAE-like clusters (both marked by high expression of *CD55* and enriched signalling for hypoxia;^48,49^ one enriched in the known stress-associated antimicrobial gene *DUOX2*^50^) have not been described previously in this model but appear to be donor, rather than treatment, driven, as they were specifically enriched in donor 2 (Extended Data Fig. 6f,g). In donor 2, we observed a cystic-like morphology in some of the organoids, and the WAE-like populations may derive therefrom^48^. Future work will be needed to substantiate the connection between these populations and organoid morphology.

On aggregate, KPT-330 increased LYZ expression (Fig. 6e) in a donor-dependent manner consistent with observations in our LYZ secretion assay and IF imaging. Furthermore, KPT-330 drives clear compositional changes, both pan-donor (decreases in cycling stem and progenitor populations, increases in quiescent progenitors and enterocytes) and donor-specific (shifts in WAE-like to DUOX2 + WAE-like) (Fig. 6f and Extended Data Fig. 6h). The populations uniquely enriched following KPT-330 treatment are also those with the highest levels of LYZ expression, including the quiescent progenitor and DUOX2 + WAE-like cells (Fig. 6g). *DUOX2* itself is a gene involved in innate barrier defence, suggesting that XPO1 inhibition via KPT-330 may induce expression of a subset of innate barrier defence genes. Finally, in addition to decreases in cycling populations and increases in innate defence gene expression and differentiation, we identified enrichment for NES-containing gene expression in the human system cycling stem populations as in the murine system. Moreover, following KPT-330 treatment, we saw the same signature compensatory increase in *XPO1* expression (Extended Data Fig. 6i). In total, the human intestinal organoid model used here demonstrated a clear effect of KPT-330 on the stem subsets as well as consistencies across species; nevertheless, its specific impact on human Paneth differentiation remains to be determined, given the original model insufficiency.

### KPT-330 induces selective expansion of Paneth cells in vivo

We next sought to validate that our framework of biological target discovery in intestinal organoids can translate to the in vivo setting. On the basis of our understanding of KPT-330-mediated XPO1 inhibition in stem-enriched murine and human organoids, we hypothesized that SINE compounds may selectively enrich the epithelium for Paneth cells in vivo. Our findings in organoids suggest that SINE treatment is independent of the niche cues of Wnt and Notch (Extended Data Fig. 2b-f), and acts specifically on cycling stem cells (which are abundant in the epithelial crypts). While XPO1 inhibition may enrich Paneth cells, goblet cells and enterocytes, by virtue of the relatively long Paneth cell lifespan^51^ we would expect a longer-term accumulation of Paneth cells in vivo relative to goblet cells or enterocytes. Additionally, because XPO1 inhibition in organoids does not expand the stem cell pool but rather re-balances patterns of differentiation, we expect an increase in Paneth cell number following SINE treatment in vivo to be restricted to the spatially constrained non-hypertrophic crypt and proportional to the initial number of cycling progenitors. This suggests that in vivo increases in Paneth cell number may be modest, yet their biological effect may be significant, and therefore a particularly sensitive method of quantification is preferable.

**Extended Data Fig. 7:**
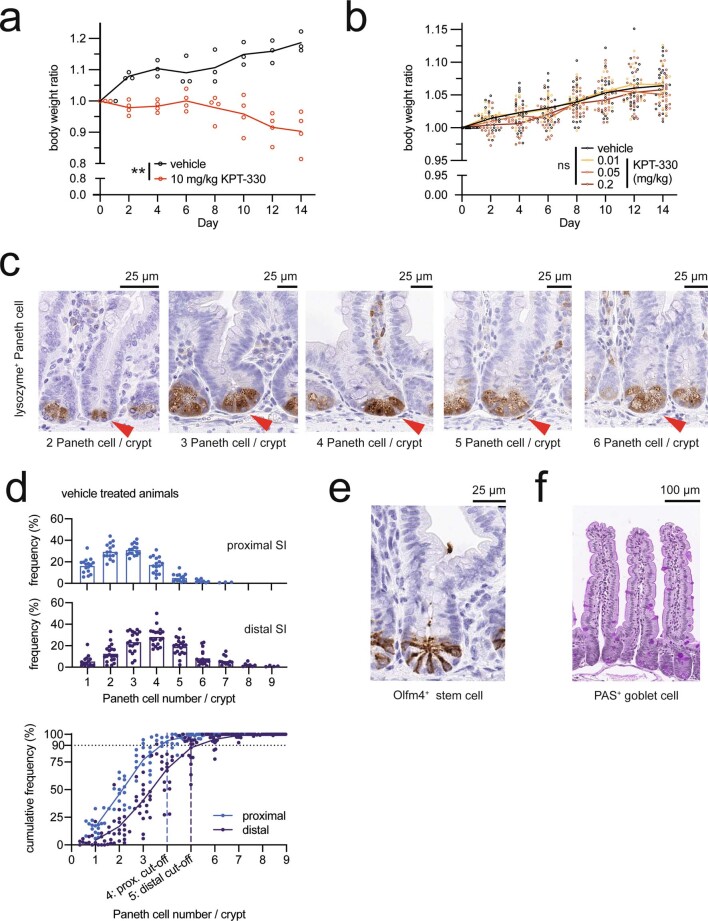
Body weight and histological images of KPT-330-treated mice. **a**, Animal body weight over 14-day study, normalized per-animal to day 0, of vehicle or 10 mg/kg KPT-330, N = 4 biological replicates followed longitudinally. Two-way ANOVA, treatment variation **p = 0.0017. **b**, Animal body weight over 14-day study, normalized per-animal to day 0, of vehicle or 0.01, 0.05 or 0.2 mg/kg KPT-330. N = 12 biological replicates (0.01 and 0.05 mg/kg), N = 20 biological replicates (vehicle and 0.2 mg/kg) followed longitudinally. Two-way ANOVA, treatment variation ns p > 0.05. **c**, Representative images of small intestinal crypt histology (counted N > 30 crypts per animal, N > 12 animals per treatment) illustrating the range of 2 to 6 Paneth cells, stained with anti-lysozyme antibody. **d**, Histograms of Paneth cell number in proximal or distal small intestine oh vehicle-treated animals. Cumulative frequency in proximal and distal small intestine of vehicle-treated animals was used for determining the cut-off value of Fig. 7C. N = 16 (vehicle and 0.2 mg/kg, proximal), N = 20 (vehicle and 0.2 mg/kg, distal), N = 12 (0.01 and 0.05 mg/kg, proximal and distal). **e**, Representative image of Olfm4^+^ stem cells in small intestinal crypts (counted N > 17 crypts per animal, N > 12 animals per treatment). **f**, Representative image of PAS^+^ goblet cells in small intestine (counted N > 15 crypts per animal, N > 12 animals per treatment). Source data

Following a similar protocol as previously reported for SINE treatment in the context of cancer^52–55^, KPT-330 was administered at a dose of 10 mg kg^−1^ via oral gavage every other day over a 2-week span in C57BL/6 wild-type mice, and body weight was monitored for any clear toxicity. Within the treatment group, we observed significant weight loss indicative of toxicity (Extended Data Fig. 7a). Given animal weight loss on the standard chemotherapeutic dosage regimen, and additional evidence that sustained dosage of SINEs adversely impacts T cell populations^56^, we sought to explore dosing regimens well below 10 mg kg^−1^, to assess whether a pro-Paneth phenotype may exist below potential toxicities.

**Fig. 7:**
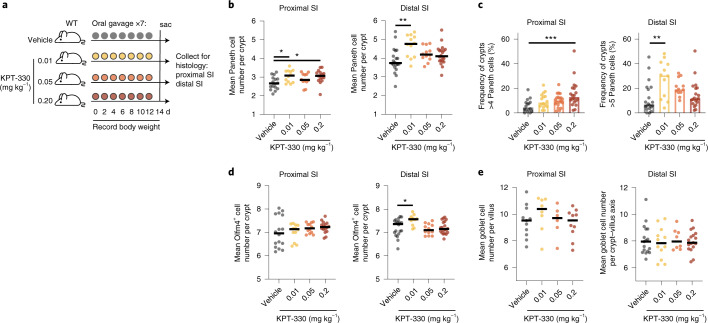
XPO1 inhibition with KPT-330 increases Paneth cell number in vivo. **a**, Design for in vivo oral gavage of KPT-330 in wild-type (WT) C57BL/6 mice. **b**, Mean Paneth cell number per crypt in proximal or distal third of small intestine, quantified by blinded histological counting. Means and individual values (representing mean of ≥30 crypt counts per biological replicate) are shown. *N* = 16 (vehicle and 0.2 mg kg^−1^, proximal), *N* = 20 (vehicle and 0.2 mg kg^−1^, distal), *N* = 12 (0.01 and 0.05 mg kg^−1^, proximal and distal). One-way ANOVA post-hoc Dunnett’s multiple comparisons test, ordered left to right: *P = 0.0175, *P = 0.0135, **P = 0.0012. **c**, Frequency of crypts with 4 or more (proximal third) or 5 or more (distal third) Paneth cells per animal across KPT-330 treatment groups, from blinded histological counts. Means and individual values (representing mean of ≥30 crypt counts per biological replicate) are shown. *N* = 16 (vehicle and 0.2 mg kg^−1^, proximal), *N* = 20 (vehicle and 0.2 mg kg^−1^, distal), *N* = 12 (0.01 and 0.05 mg kg^−1^, proximal and distal). One-way ANOVA post-hoc Dunnett’s multiple comparisons test: ***P = 0.0007, **P < 0.0017. **d**, Mean Olfm4^+^ stem cell number per crypt in proximal or distal third of small intestine, quantified by blinded histological counting. Means and individual values (representing mean of ≥17 crypt counts per biological replicate) are shown. *N* = 16 (vehicle and 0.2 mg kg^−1^, proximal), *N* = 20 (vehicle and 0.2 mg kg^−1^, distal), *N* = 12 (0.01 and 0.05 mg kg^−1^, proximal and distal). One-way ANOVA post-hoc Dunnett’s multiple comparisons test: *P = 0.0112. **e**, Mean PAS^+^ goblet cell number per villus in proximal or distal third of small intestine, quantified by blinded histological counting. Means and individual values (representing mean of ≥15 villus counts per biological replicate) are shown. *N* = 11, 8, 6, 10 (vehicle, 0.01, 0.05. 0.2 mg kg^−1^, proximal), *N* = 17, 12, 8, 17 (vehicle, 0.01, 0.05. 0.2 mg kg^−1^, distal). Source data

We repeated the 2-week study with oral gavage of KPT-330 every other day at doses corresponding to 50-fold (0.2 mg kg^−1^), 200-fold (0.05 mg kg^−1^) and 1,000-fold (0.01 mg kg^−1^) decrease in the 10 mg kg^−1^ dose conventionally used in a cancer setting. Because Paneth cell number and quality are known to physiologically change along the length of the small intestine, and diseases associated with Paneth cells most frequently present distally^57^, we sought to profile how XPO1 inhibition may differentially affect the proximal and distal small intestine. We tracked animal weight every other day and collected the proximal and distal thirds of the small intestine at day 14 for histological quantification of Paneth, stem and goblet populations (Fig. 7a). In this lower-dose regimen, we observe no significant changes in animal weight, suggesting that the doses are outside the gross toxicity range (Extended Data Fig. 7b). Paneth cells were counted within well-preserved crypts, with at least 30 crypts quantified per animal (representative images in Extended Data Fig. 7c), and the counts were averaged. Within this lower-dose regimen, we observe significant increases in Paneth cell abundance both in the proximal and distal small intestine at doses of 0.01 mg kg^−1^, and proximally at 0.2 mg kg^−1^ (Fig. 7b). To demonstrate the significance of this increase, we identified the 90th percentile abundance of Paneth cells per crypt in vehicle animals (4 per crypt proximally and 5 per crypt distally) (Extended Data Fig. 7d) and calculated the fraction of crypts with a greater number of Paneth cells than the 90th percentile cutoff for each KPT dose. In the proximal small intestine, 0.2 mg kg^−1^ nearly tripled this fraction (15.5% vs vehicle 5.8%), while in the distal small intestine, 0.01 mg kg^−1^ more than doubled this fraction (27.9% vs 12.2%). We additionally quantified the abundance of Olfm4+ stem cells as well as PAS+ goblet cells within the same animals to ascertain whether the effect of SINE treatment was restricted to the Paneth cell compartment (representative images in Extended Data Fig. 7e,f). We observe a significant increase in Olfm4+ stem cells within the distal small intestine (SI) at doses of 0.01 mg kg^−1^, corresponding to the group with the greatest increase in Paneth cells (Fig. 7d), suggesting a potential expansion of the stem cell niche commensurate with increased Paneth cell abundance. We did not identify any significant changes in the developmentally related goblet cell population (Fig. 7e). In total, these data suggest that SINE treatment may be a meaningful approach to specifically increase Paneth cell abundance in vivo, and further validates our framework for using models of organoid differentiation in small-molecule screening.

## Discussion

Here we demonstrate that by employing phenotypic small-molecule screening in a physiologically motivated organoid model, we can uncover biological targets and clinically relevant small molecules that translate to in vivo and inform cross-species (mouse to human) tissue stem cell biology, without previous knowledge of that biological target’s function in vivo. Further, this approach to small-molecule phenotypic screening enables a specific, functional readout in a dynamic and heterogeneous organoid model, and may be suited to uncover targets not readily amenable to genetic perturbation (for example *Xpo1*, a gene essential to cell survival through its role in mitosis). Our approach provides perturbation capacity nearly two orders of magnitude greater than existing examples of non-genetic perturbations in organoid models, thereby enabling screens within the space of annotated small-molecule libraries and empowering biological target discovery.

By using a model that focuses on differentiation to a specific lineage (the Paneth cell), we are able to resolve a pathway and compounds that direct ISC fate decisions to drive subtle but significant effects on the in vivo tissue. We identify a series of compounds known to inhibit the nuclear exporter XPO1, acting on cycling ISCs by inducing a programme of stress response and decreased mitogen signalling. This ISC response re-balances self-renewal and differentiation towards Paneth cell differentiation. Recent work on mitogen and stress response control of re-entry into the cell cycle may provide context on the necessity of overlap of these two responses in directing decisions of fate commitment^58^. Transitions between quiescence and proliferation within the ISC niche have roles in tissue homoeostasis and regeneration. Quiescent cells serve as reserve populations that upon injury of cycling stem cells, will re-establish progenitors and maintain homoeostatic tissue regeneration^59,60^. Further, a transient quiescent ISC state facilitates secretory enteroendocrine cell differentiation^46^, and may explain why we see synergistic Paneth cell enrichment with a combination of ERK and XPO1 inhibition. Furthermore, ERK inhibition may affect differentiation by either suppression of enterocyte differentiation^61^ or augmentation of Wnt signalling^62^. Additionally, we see that the pan-epithelial stress response induced by KPT-330-mediated XPO1 inhibition in vitro involves the AP-1 pathway, which also appears to play a role in Paneth differentiation and may be mediated via transcriptional changes in Atf3. in vivo, Atf3 is implicated in the regulation of stress responses in disease of the barrier tissue^42,63^, where XPO1 may be one way to access these observed responses for therapeutic use.

Critical aspects from our murine organoid study appear to carry over to the human organoid model. We observe that KPT-330-mediated XPO1 inhibition clearly suppresses cell cycle activity and encourages differentiation and expression of innate defence genes (*LYZ*, *DUOX2*). It appears that KPT-330-mediated XPO1 inhibition induces a unique quiescent progenitor population in the human organoids, which may have resemblance to a pro-secretory progenitor known to be important in the murine model^46^. It is also clear that because the human model lacks Paneth cells, direct observation of differentiation and further screening for modulators of Paneth cells is limited. Future refinement of the human organoid model would empower more definitive conclusions about XPO1’s role in human Paneth cell development. Finally, understanding drivers of donor-to-donor variability in the human setting also poses a challenge for the field moving forward.

While we have demonstrated that KPT-330-mediated XPO1 inhibition in vivo significantly increases the abundance of Paneth cells within the small intestinal crypts, we believe there are key mediators that may explain heterogeneity of biological potency along the small intestine, and may be investigated to enhance effect in future studies. Our in vitro work suggests that KPT-330-mediated XPO1 inhibition induces differentiation, we would therefore not expect a gross remodelling of crypt architecture, which is an inherently constrained space in vivo, having an average of 3.5 to 4 Paneth cells, and thus would anticipate that increases in Paneth cell number would be modest and in line with tissue microarchitecture along the gastrointestinal tract. In fact, we feel that the increase of an additional 0.5–1 Paneth cell per crypt that we observe in vivo is in favour of the specific and significant effect of the identified agent. We further note that this demonstration specifically increases Paneth abundance in vivo without inducing crypt hypertrophy or targeting potentially oncogenic pathways, avoiding concerns with previous agents shown to modulate in vivo Paneth abundance (Wnt activators and Notch inhibitors).

We also strongly believe that future study outside the scope of the present work is warranted to better elucidate the role of XPO1 and XPO1 inhibitors in modulating adult epithelial stem cell fate commitment. Our present evidence for XPO1’s role in mediating intestinal stem cell differentiation in a pro-Paneth manner is known only by chemical inhibition, and there may exist yet undescribed off-target effects of the inhibitors tested here that could inform the observations leading to our proposed mechanism. Future work to explore the biological role of XPO1 in intestinal stem cell differentiation through genetic means, such as the use of an XPO1 C528S mutant^64^ in an organoid or in vivo, are critical next steps to confirming a mechanistic role for XPO1. Additionally, as our knowledge of the in vivo stem cell niche improves, organoid models can be refined in an iterative fashion, further enhancing model fidelity and increasing the probability of compound translation from in vitro to in vivo conditions.

This approach – employing a physiologically motivated set of cues to modulate ISC differentiation and layering on a screen for unreported regulators of that differentiation at scale – may be further applied both within the small intestine and more broadly across adult barrier tissues to modulate tissue composition through unreported molecular targets and associated small molecules. For example, this same framework may be applied in the context of enteroendocrine cell development within the small intestine to explore ways in which hormone secretion, including that of Glp-1, may be modulated or in the context of goblet cell development to identify agents that may transiently enhance mucus production. While establishing the appropriate model and screening assay for a study of enteroendocrine or goblet differentiation is not trivial, it should be possible based on the approach we have demonstrated here. Additionally, a small-molecule approach may offer the benefit of transient control of tissue composition, which may be reversed with removal of the agent. Overall, we provide a framework to construct organoid models of lineage-specific differentiation that can uncover pathways regulating differentiation and reveal compounds controlling barrier tissue composition.

## Methods

### Murine crypt isolation and organoid culture

Small intestinal crypts were isolated under animal protocols approved by the Massachusetts Institute of Technology (MIT) Committee on Animal Care (CAC). Proximal and/or distal small intestines were isolated from wild-type C57BL/6 mice of both sexes, aged between 1 and 6 months in all experiments. Small intestinal crypts were isolated as previously described^23^. Briefly, the small intestine was collected, opened longitudinally and washed with ice-cold Dulbecco’s phosphate buffered saline without calcium chloride and magnesium chloride (PBS0) (Sigma-Aldrich) to clear the luminal contents. The tissue was cut into 2–4 mm pieces with scissors and washed repeatedly by gently pipetting the fragments using a 10 ml pipette until the supernatant was clear. Fragments were rocked on ice with crypt isolation buffer (2 mM EDTA in PBS0; Life Technologies) for 30 min. After isolation buffer was removed, fragments were washed with cold PBS0 by pipetting up and down to release the crypts. Crypt-containing fractions were combined, passed through a 70 μm cell strainer (BD Bioscience), and centrifuged at 300 r.c.f. for 5 min. The cell pellet was resuspended in basal culture medium (2 mM GlutaMAX (Thermo Fisher) and 10 mM HEPES (Life Technologies) in advanced DMEM/F12 (Invitrogen)) and centrifuged at 200 r.c.f. for 2 min to remove single cells. Crypts were then cultured in a Matrigel culture system (described below) in small intestinal crypt medium (100X N2 supplement (Life Technologies), 100X B27 supplement (Life Technologies), 1 mM *N*-acetyl-l-cysteine (Sigma-Aldrich) in basal culture medium) supplemented with differentiation factors at 37 °C with 5% CO_2_. Penicillin/streptomycin (100X) was added for the first 4 d of culture post-isolation only.

Small intestinal crypts were cultured as previously described^23^. Briefly, crypts were resuspended in basal culture medium at a 1:1 ratio with Corning Matrigel membrane matrix–GFR (Thermo Fisher) and plated at the centre of each well of 24-well plates. Following Matrigel polymerization, 500 μl of crypt culture medium (ENR + CV) containing growth factors EGF (50 ng ml^−1^, Life Technologies), Noggin (100 ng ml^−1^, PeproTech) and R-spondin 1 (500 ng ml^−1^, PeproTech) and small molecules CHIR99021 (3 μM, LC Laboratories or Selleck Chem) and valproic acid (1 mM, Sigma-Aldrich) was added to each well. ROCK inhibitor Y-27632 (10 μM, R&D Systems) was added for the first 2 d of ISC culture only. The cell culture medium was changed every other day. After 4 d of culture, crypt organoids were expanded as and enriched for ISCs under the ENR + CV condition. Expanding ISCs were passaged every 4–6 d in the ENR + CV condition.

After 2–6 d of culture under ENR + CV condition, ISCs were differentiated to Paneth cells. Briefly, ISC culture gel and medium were homogenized via mechanical disruption and centrifuged at 300 r.c.f. for 3 min at 4 °C. The supernatant was removed and the pellet resuspended in basal culture medium repeatedly until the cloudy Matrigel was almost gone. On the last repeat, the pellet was resuspended in basal culture medium, the number of organoids counted, and the suspension centrifuged at 100 r.c.f. for 1 min at 4 °C. The cell pellet was resuspended in basal culture medium at a 1:1 ratio with Matrigel and plated at the centre of each well of 24-well plates (~100–250 organoids per well). Following Matrigel polymerization, 500 μl of crypt culture medium (ENR + CV) was added to each well. The cell culture medium was changed every 2–4 d depending on seeding density.

### Human crypt isolation and organoid culture

Excess surgical tissue samples from adult human duodenum were collected for organoid culture in accordance with Massachusetts General Hospital Institutional Review Board (IRB) guidance under Mass General Brigham Protocol 2010P000632. De-identified human donor tissue was collected following medically indicated bulk surgical resection via MGH Pathology as excess tissue. Donors of both sexes, aged between 58–74 years, presented with pathologies unrelated to the duodenum. Crypts were isolated from bulk resections as follows. Bulk resections were cut into approximately 0.25 cm sections from the epithelial surface, and washed in PBS0 repeatedly by gently pipetting the fragments using a 10 ml pipette until the supernatant was clear. Fragments were rocked on ice with crypt isolation buffer (10 mM EDTA, 10 mM HEPES, 2% FCS in PBS0) for 30 min. After isolation buffer was removed, fragments were washed with cold PBS0 by vigorous shaking to release the crypts. This process was repeated with reserved crypt-laden supernatant fractions 4–6 times or until supernatant was free of intact crypts (visual inspection). Crypt-containing fractions were combined, passed through a 100 μm cell strainer (BD Bioscience), and centrifuged at 300 r.c.f. for 5 min. The crypt pellet was resuspended in basal culture medium (2 mM GlutaMAX (Thermo Fisher) and 10 mM HEPES (Life Technologies) in advanced DMEM/F12 (Invitrogen)) and centrifuged at 200 r.c.f. for 2 min to remove single cells. Crypts were then cultured in a Matrigel culture system (described previously). Organoids were cultured and passaged as described for murine organoids every 6–8 d in Matrigel domes with established media conditions meant to recapitulate a stem cell-enriched condition^47^. Organoid culture media contained recombinant EGF (Thermo Fisher), FGF2 (Thermo Fisher), IGF1 (Peprotech), Gastrin (Sigma Millipore) and TGF-b inhibitor A83-01 (Tocris Bioscience) with 50% conditioned medium of L-cell line secreting Wnt3a, R-spondin3 and Noggin (L-WRN CM) supplemented with 10 μmol l^−1^ Y-27632 (Tocris Bioscience). L-WRN CM was prepared from L-WRN (ATCC; CRL-3276) as described previously^65^. L-WRN (50%) is a 1:1 mixture of 100% L-WRN and primary culture medium. Primary culture media consist of advanced DMEM/F12, penicillin/streptomycin, GlutaMAX (all from Thermo Fisher), and FBS (20%). Organoid samples grown in culture over varying periods were either maintained and passaged or treated with 160 nM KPT-330 for 6 d before assay, with media changes every other day.

### High-throughput screening

For 384-well plate high-throughput screening, ISC-enriched organoids were passaged and split into single cells with TyrpLE (Thermo Fisher) and cultured for 2–3 d in ENR + CVY (Y: Y-27632 at 10 μM) before transfer to a ‘2.5D’ 384-well plate culture system. To prepare for ‘2.5D’ plating, cell-laden Matrigel and media were homogenized via mechanical disruption and centrifuged at 300 r.c.f. for 3 min at 4 °C. The supernatant was removed and the pellet washed and spun in basal culture medium repeatedly until the cloudy Matrigel above the cell pellet was gone. On the final wash, the pellet was resuspended in basal culture medium, the number of organoids counted, and the cell pellet resuspended in ENR + CD medium at ~7 clusters per μl. Plates (384-well) were first filled with 10 μl 70% Matrigel (30% basal media) coating in each well using a Tecan Evo 150 liquid handling deck, and allowed to gel at 37 °C for 5 min. Then 30 μl of cell-laden media was plated at the centre of each well of 384-well plates with the liquid handler, and the plates were spun down at 100 r.c.f. for 2 min to embed organoids on the Matrigel surface. Compound libraries were pinned into prepped cell plates using 50 nl pins into 30 μl media per well. Cells were cultured at 37 °C with 5% CO_2_ for 6 d in ENR + CD medium supplemented with the tested compounds, with media change at 3 d. On day 6, lysozyme secretion and cell viability were assessed using lysozyme assay kit (EnzChek) and CellTiter-Glo 3D (CTG 3D) cell viability assay (Promega), respectively, according to the manufacturers’ protocols. Briefly, screen plates were washed 3× with FluoroBrite basal media (2 mM GlutaMAX and 10 mM HEPES in FluoroBrite DMEM (Thermo Fisher)) using a BioTek 406 plate washer with 10 min incubations, followed by a 1 min centrifugation at 200 r.c.f. to settle media between washes. After removal of the third wash, 30 μl of non-stimulated FluoroBrite basal media was added to each screen well using a Tecan Evo 150 liquid handling deck from a non-stimulated treatment master plate, and plates were incubated for 30 min at 37 °C. After 30 min, the top 15 μl of media from each well of the screen plate was transferred to a non-stimulated LYZ assay plate containing 15 μl of 20X DQ LYZ assay working solution using a Tecan Evo 150 liquid handling deck. The non-stimulated LYZ assay plate was covered, shaken for 10 min, incubated for 50 min at 37 °C, then fluorescence measured (shaken for 10 s; 494 mm/518 nm) using a Tecan M1000 plate reader. After media transfer to the non-stimulated LYZ assay plate, the remaining media were removed from the screen plate and 30 μl of stimulated FluoroBrite basal media (supplemented with 10 μM CCh) was added to each screen well using a Tecan Evo 150 liquid handling deck from a stimulated treatment master plate, and plates were incubated for 30 min at 37 °C. After 30 min, the top 15 μl of media from each well of the screen plate was transferred to a stimulated LYZ assay plate containing 15 μl of 20X DQ LYZ assay working solution using a Tecan Evo 150 liquid handling deck. The stimulated LYZ assay plate was covered, shaken for 10 min, incubated for 50 min at 37 °C, then fluorescence measured (shaken for 10 s; 494 mm/518 nm) using a Tecan M1000 plate reader. Finally, 8 μl of CTG 3D was added to each well of the screen plate, which was shaken for 30 min at room temperature, then luminescence read (shaken for 10 s; integration time 0.5–1 s) to measure ATP.

Primary screens were performed using the Target-Selective Inhibitor Library (Selleck Chem). Assays were performed in triplicate using 4 compound concentrations (0.08, 0.4, 2 and 10 μM).

### Screen analysis

Analysis of all screen results was performed in R. Results (excel or.csv files) were converted into a data frame containing raw assay measurements corresponding to metadata for plate position, treatments, doses, cell type and stimulation. Raw values were log_10_ transformed, then a locally estimated scatterplot smoothing (LOESS) normalization was applied to each plate and assay to remove systematic error and column/row/edge effects using the formula:^66^

1 $$\widehat {x_{ij}} = x_{ij} - \left(\mathrm{loess.fit}_{ij} - \mathrm{median}\left( {\mathrm{loess.fit}_{ij}} \right)\right),$$

where $\widehat {x_{ij}}$ is the LOESS fit result, $x_{ij}$ is the log_10_ transformed value at row *i* and column *j*, and $\mathrm{loess.fit}_{ij}$ is the value from LOESS smoothed data at row *i* and column *j* calculated using R loess function with span 1.

Following LOESS normalization, a plate-wise fold change (FC) calculation was performed on each well to normalize plates across the experiment. This was calculated by subtracting the median of the plate (as control) from the LOESS normalized values:

2 $$\mathrm{FC}_{ij} = \widehat {x_{ij}} - \mathrm{median}\left( {\widehat {x_{ij}}} \right).$$

Replicate strictly standardized mean difference (SSMD) was used to determine the statistical effect size of each treatment in each assay (treatment and dose grouped by replicate, *n* = 3) relative to the plate, using the formula for the robust uniformly minimal variance unbiased estimate (UMVUE):^67^

3 $$\mathrm{SSMD} = \frac{{\Gamma (\frac{{n - 1}}{2})}}{{\Gamma (\frac{{n - 2}}{2})}}\sqrt {\frac{2}{{n - 1}}} \frac{{\overline {d_i} }}{{\sqrt {w_is_i^2 + w_0s_0^2} }},$$

where $\overline {d_i}$ and *s*_*i*_ are respectively the sample mean and standard deviation of *d*_*ij*_s where *d*_*ij*_ is the FC for the *i*th treatment on the *j*th plate. $\Gamma ( \cdot )$ is a gamma function. $s_0^2$ is an adjustment factor equal to the median of all $s_i^2$s to provide a more stable estimate of variance. *w*_*i*_ and *w*_*0*_ are weights equal to 0.5 with the constraint of *w*_*i*_ + *w*_*0*_ = 1. *n* is the replicate number.

Mean FC (the arithmetic mean of all samples grouped by treatment and dose across replicates) was used to determine the *z*-score for each treatment and dose with the formula:

4 $$z = \frac{{\mathrm{meanFC}}}{{\mathrm{SD}_{\rm{pop}}}},$$

where SD_pop_ is the standard deviation of all mean FCs.

All calculated statistics were combined in one finalized data table and exported as a.csv file for hit identification. A primary screen ‘hit’ was defined as having SSMDs for both LYZ assays greater than the optimal critical value ($\beta _{\alpha _1}$ = 0.997) and being in the top 10% of a normal distribution of FC values for both assays with a *z*-score cutoff >1.282. $\beta _{\alpha _1}$ was determined by minimizing the false positive (FPL) and false negative (FNL) levels for upregulation SSMD-based decisions by solving for the intersection of the formulas:^67^

5 $$F_{t\left( {n - 1,\sqrt n \beta _2} \right)}\left( {\frac{{\beta _{\alpha _1}}}{k}} \right) = 1 - \mathrm{FPL}$$

and

6 $$\mathrm{FNL} = F_{t\left( {n - 1,\sqrt n \beta _1} \right)}\left( {\frac{{\beta _{\alpha _1}}}{k}} \right),$$

where

7 $$k = \sqrt {\frac{1}{n}}$$

and $F_{t\left( {n - 1,\sqrt n \beta } \right)}$ is the cumulative distribution function of non-central *t*-distribution $t(n - 1,\sqrt n \beta )$, *n* is the number of replicates, $\beta _2$ is an SSMD bound for FPL of 0.25 (at least very weak effect) and $\beta _1$ is an SSMD bound for FNL of 3 (at least strong effect).

Hit treatments were thus selected to have a well-powered statistical effect size as well as a strong biological effect size. Optimal dose per hit treatment was determined by SSMD for both LYZ assays.

### Secondary lysozyme secretion assay screen

Confirmatory secondary screening with primary hits was performed using the above 384-well plate method. The screen was conducted with 4-plate replicates with a base media of ENR+CD. Media was supplemented with compound at day 0 and day 3 (*n* = 8 well replicates per dose) at 4 different doses: twofold above, twofold below and fourfold below the optimal final dose for each respective treatment. Additionally, each plate carried a large number of ENR+DMSO or ENR+CD+DMSO (vehicle) control wells (*n* = 100 for ATP, and *n* = 25 for LYZ.NS and LYZ.S) for robust normalization. ATP, non-stimulated lysozyme activity and CCh-stimulated lysozyme activity were again measured and the collected data were again processed in a custom R-script per primary screen, with slight modification. Values were log_10_ transformed, and a plate-wise FC was calculated for each well on the basis of the median value of ENR+CD+DMSO (vehicle) control wells to normalize plate to plate variability. The following formula was used:

8 $$\mathrm{FC}_{ij} = x_{ij} - \mathrm{median}\left( {x_{\mathrm{POS}}} \right),$$

where $x_{ij}$ is the log_10_ transformed value at row *i* and column *j*, and $x_{\mathrm{POS}}$ are the values of the positive control wells. For the ATP assay, all vehicle-only wells were used as the control. For the LYZ.NS assay, non-stimulated vehicle-only wells were used. For the LYZ.S assay, vehicle-only wells that were non-stimulated in the LYZ.NS assay then stimulated in the LYZ.S assay were used.

Once normalized, the replicate SSMD was calculated using equation (3) to quantify statistical effect size, with 8 replicate differences taken relative to the respective plate ENR+DMSO or ENR+CD+DMSO median value. A primary hit was considered validated when SSMDs for both LYZ assays were greater than the optimal critical value ($\beta _{\alpha _1}$) of 0.889. $\beta _{\alpha _1}$ was determined using equation (5), with an FPL error of 0.05 for a more stringent cutoff; FNL was not considered. Optimal doses were chosen for treatments with multiple validated doses by taking the most potent (highest mean fold change relative to ENR+CD control) dose in both LYZ assays.

### Lysozyme secretion assay

ISC-enriched organoids in 3D Matrigel culture were passaged to a 48- or 96-well plate and cultured with ENR or ENR+CD media containing DMSO or each drug for 6 d. DMSO- or drug-containing media were changed every other day. On day 6, cells were washed with basal media twice and treated with basal media with or without 10 μM carbachol for 3 h in a CO_2_ incubator at 37 °C. Conditioned media was collected and used for lysozyme assay (Thermo Fisher, E-22013) following the manufacturer’s instruction. The fluorescence was measured using excitation/emission of 485/530 nm. CTG 3D Reagent was added afterward, and the cell culture plate was incubated on an orbital shaker at RT for 30 min to induce cell lysis and to stabilize the luminescent signal. The solution was replaced to a 96-well white microplate, and luminescent signals were measured by a microplate reader (infinite M200, Tecan). The standard curve was prepared by diluting recombinant ATP (Promega, P1132). For both assays, a polynomial cubic curve was fitted to a set of standard data, and each sample value was calculated on the Microsoft Excel.

### Flow cytometry

ISC-enriched organoids in 3D Matrigel culture were passaged to a 24- or 48-well plate and induced to differentiate for 6 d by ENR+CD media containing DMSO or each drug indicated in the figures. DMSO- or drug-containing media were changed every other day. On day 6, cells were washed twice with basal media, then collected from Matrigel by mechanical disruption in TrypLE Express (Thermo Fisher, 12605010) to remove Matrigel and dissociate organoids to single cells. After vigorous pipetting and incubation at 37°C for 15 min, the cell solution was diluted twice with basal media and centrifuged at 300 r.c.f. for 3 min. The cell pellet was resuspended in FACS buffer (PBS containing 2% FBS) and replaced into a 96-well clear round-bottom ultra-low attachment microplate (Corning, 7007). The cell solution was centrifuged again at 300 r.c.f. for 3 min at 4 °C to pellet the cells. Cells were stained with Zombie-violet dye (BioLegend, 423113, 1:100) at 100X for viability staining for 20 min at r.t. in the dark. After centrifugation for 3 min at 300 r.c.f., cells were fixed in fixation buffer (FACS buffer containing 1% formaldehyde (Thermo Fisher, 28906)) for 15 min on ice in the dark. Cells were centrifuged again for 3 min at 300 r.c.f. and blocked with staining buffer (FACS buffer containing 0.5% Tween 20 (Sigma, P2287)) for 15 min at r.t. in the dark. Cells pelleted by centrifugation for 3 min at 300 r.c.f. were stained with FITC-conjugated anti-lysozyme antibody (Dako, F0372, 1:100) and APC-conjugated anti-CD24 antibody (Biolegend, 138505, 1:100) at 100X for 45 min at r.t. in the dark. The cell pellet was washed once with FACS buffer, resuspended in FACS buffer and filtered through a 5 ml test tube with cell strainer snap cap (Corning, 352235). Flow cytometry was performed using an LSR Fortessa (BD; Koch Institute Flow Cytometry Core at MIT). Flow cytometry data were analysed using FlowJo X v10.6.1 software.

### Western blotting

Organoid-containing gel was homogenized in basal medium and centrifuged at 300 r.c.f. for 3 min. The organoid pellet was lysed with ice-cold Pierce IP lysis buffer (Thermo Fisher, 87787) containing EDTA-free Halt protease inhibitor cocktail (Thermo Fisher, 87785) and incubated on ice for 20 min. The lysate was centrifuged at 17,000 r.c.f. for 10 min, and the supernatant was combined with NuPAGE LDS sample buffer (Thermo Fisher, NP0007). Protein concentration was determined by Pierce 660 nm protein assay (Thermo Fisher, 22660) and normalized to the lowest concentration among each sample set. Samples were incubated at 70°C for 10 min and resolved by SDS–PAGE using NuPAGE 4–12% Bis-Tris protein gels (Thermo Fisher), followed by electroblotting onto Immun-Blot PVDF Membrane (Biorad, 1620174) using Criterion blotter with plate electrodes (Biorad, 1704070). The membranes were blocked with 2% blotting-grade blocker (Biorad,1706404) in TBS-T (25 mM Tris–HCl, 140 mM NaCl, 3 mM potassium chloride and 0.1% Tween 20) and then probed with appropriate antibodies, diluted in TBS-T containing 2% BSA (Sigma, A7906) and 0.05% sodium azide (Sigma, 71289). The primary antibody against lysozyme was purchased from Abcam (ab108508 1:2000). HRP-linked anti-rabbit IgG antibodies were purchased from Cell Signalling Technology (7074, 1:2,000). Chemiluminescent signals were detected by LAS4000 (GE Healthcare) using Amersham ECL Select western blotting detection reagent (GE Healthcare, 45-000-999), and total protein signals were obtained by Odyssey imaging system (LI-COR Biosciences) using REVERT total protein stain kit (LI-COR Biosciences, 926-11010).

### Immunofluorescent imaging

For immunofluorescence staining of organoids, intestinal organoids in Matrigel were fixed with 4% paraformaldehyde, then transferred to centrifuge tubes. After washing with PBS, the isolated organoids were permeabilized with 1% Triton X, followed by incubation with blocking buffer (1% BSA + 3% Donkey Serum + 0.2% Triton X in PBS) at r.t. The organoids were then stained with primary antibodies and fluorescent dye-labelled secondary antibodies, as well as with 4′,6-diamidino-2-phenylindole (DAPI). Slides were covered with VECTASHIELD mounting media (VECTOR). The following primary and secondary antibodies were used for the staining: rabbit anti-lysozyme (Thermo Fisher, RB-372-A, 1:1,000), rat anti-E-cadherin (Thermo Fisher, 13-1900, 1:1,000), and Alexa Fluor 488 and 568 secondary antibodies (Thermo Fisher A21208, A10042, 1:1,000). Images were acquired with a confocal laser scanning microscope (Nikon Eclipse 90i) with the following acquisition settings: DAPI exposure time 2 ms, contrast gain 0; FITC (for E-cadherin) exposure time 39 ms, contrast gain 0; TRITC (for lysozyme) exposure time 30 ms, contrast gain 0. For the analysis of lysozyme+ cells per organoid area, the number of counted lysozyme+ cells were normalized to the measured organoid surface area. Fiji v2.0 was used for quantification of lysozyme+ cells.

### Animal study

All animal studies were performed under animal protocols approved by the MIT CAC. Wild-type C57BL/6NCrl male mice (8–10-week-old, 027) were purchased from Charles River. Mice were housed under 12 h light/dark cycle, provided food and water ad libitum, and kept in a 20–22 °C and 30–70% humidity environment. KPT-330 (0.01, 0.05, 0.2 or 10 mg kg^−1^) were injected orally using a disposable gavage needle (Cadence Science, 9921) at 10 μl g^−1^ weight. KPT-330 was dissolved in DMSO initially and further diluted in sterile PBS containing Pluronic F-68 non-ionic surfactant (Gibco, 24040032) and polyvinylpyrrolidone (PVP, Alfa Aesar, A14315, average M.W. 58,000); the final concentration of DMSO is 2%, Pluronic 0.5% and PVP 0.5%. KPT-330 was administered every other day for 2 weeks, for a total of 7 injections (days 0, 2, 4, 6, 8, 10, 12), and mice were killed at day 14.

### Histology

The SI was collected from mice and divided into three parts. Only proximal and distal SI were kept in PBS, and medial SI was discarded. Each SI was opened longitudinally and washed in PBS. SI was rolled using the Swiss-rolling technique and incubated in 10% neutral buffered formalin (VWR, 10790-714) for 24 h at r.t. Fixed tissues were embedded in paraffin and 4 μm sections were mounted on slides. For immunohistochemistry, slides were deparaffinized, antigen retrieved using heat-induced epitope retrieval at 97 °C for 20 min with citrate buffer pH 6, and probed with appropriate antibodies, followed by 3,3′-Diaminobenzidine (DAB) staining. An antibody against lysozyme was purchased from Abcam (ab108508, 1:2,000), Ki67 from BD Biosciences (550609 1:40) and Olfm4 from Cell Signalling Technology (39141, 1:1,000). For McManus periodic acid Schiff (PAS) reaction, slides were deparaffinized, oxidized in periodic acid and stained with Schiff reagent (Poly Scientific, s272), followed by counterstaining with Harris hematoxylin. Slides were scanned using an Aperio slide scanner (Leica) and cells were counted on an Aperio eSlide Manager. Slides were blinded and randomized before counting, and all cell types were counted in all well-preserved crypts along the longitudinal crypt–villus axis (Paneth cell, ≥30 crypts; Olfm4+ cell, ≥17 crypts; goblet cell, ≥15 villi, per sample). For the goblet cell images, the samples that included <15 well-preserved crypt–villus axes were excluded, which was predetermined.

### Murine and human scRNA-seq and alignment

A single-cell suspension was obtained from murine organoids cultured under either ENR+CD or ENR+CD+160 nM KPT-330 for the differentiation time course as detailed in Fig. 2a, or human organoids treated with 160 nM KPT-330 as detailed in Fig. 6a. For both, organoids at each sampling were collected from 4–6 pooled Matrigel domes, totalling >1,000 organoids per sample. Excess Matrigel was removed per previously described washing protocol, and organoids were resuspended in TrypLE at 37 ºC for 15 min, with vigorous homogenization through a p200 pipette tip every 5 min. After 15 min, the suspension was passed through a 30 uM cell strainer twice and counted under bright-field microscopy with trypan blue staining for viable single cells. For human organoid scRNA-seq, antibody-based cell hashing was performed, with all samples pooled following labelling and three washes in FACS buffer to remove excess antibody. Each sample was manually counted to equally weight in cell pools, and then the pool was split and processed as four identical samples.

We utilized Seq-Well S^3^ for massively parallel scRNA-seq, for which full methods are published^36^ and made available on the Shalek Lab website (www.shaleklab.com). Briefly, ~15,000–20,000 cells were loaded onto a functionalized-polydimethylsiloxane (PDMS) array preloaded with ~80,000 uniquely barcoded mRNA capture beads (Chemgenes; MACOSKO-2011-10). After cells had settled into wells, the array was then sealed with a hydroxylated polycarbonate membrane with pore size of 10 nm, facilitating buffer exchange while confining biological molecules within each well. Following membrane-sealing, buffer exchange across the membrane permitted cell lysis, mRNA transcript hybridization to beads and bead removal before proceeding with reverse transcription. The obtained bead-bound complementary DNA (cDNA) product then underwent Exonuclease I treatment (New England Biolabs; M0293M) to remove excess primer before proceeding with second strand synthesis.

Following Exonuclease I treatment, the beads were mixed with 0.1 M NaOH for 5 min at r.t. to denature the mRNA–cDNA hybrid product on the bead. Second strand synthesis was performed with a mastermix consisting of 40 ul 5x maxima RT buffer, 80 ul 30% PEG8000 solution, 20 ul 10 mM dNTPs, 2 ul 1 mM dn-SMART oligo, 5 ul Klenow Exo- and 53 ul DI ultrapure water, with the mastermix being added to the beads and incubated for 1 h at 37 °C with end-over-end rotation. After the second strand synthesis, PCR amplification was performed using KAPA HiFi PCR Mix (Kapa Biosystems, KK2602). Specifically, a 40 ul PCR Mastermix consisting of 25 ul KAPA 5X Mastermix, 0.4 ul 100 uM ISPCR oligo and 14.6 ul nuclease-free water was combined with 2,000 beads per reaction. Following PCR amplification, whole transcriptome products were isolated through two rounds of SPRI purification using Ampure Spri beads (Beckman Coulter) at both 0.6X and 0.8X volumetric ratio and quantified using a Qubit. For the antibody hashed human organoid samples, the first SPRI supernatant was retained and subjected to an additional SPRI at 2X final volumetric ratio and quantified using a Qubit. The hashing library then went through a round of step-up PCR to append sequencing handles and indices, followed by a final 1.6X volumetric ratio SPRI before final pooling with the mRNA library (below).

Sequencing libraries were constructed from whole transcriptome product using the Nextera Tagmentation method on a total of 800 pg of pooled cDNA library per sample. Tagmented and amplified sequences were purified through two rounds of SPRI purification (0.6X and 0.8X volumetric ratios) yielding library sizes with an average distribution of 500–750 base pairs in length as determined using the Agilent hsD1000 screen tape system (Agilent Genomics). Murine organoid arrays were sequenced within multi-sample pools on an Illumina NovaSeq through the Broad Institute walk-up sequencing core. Human organoid arrays were sequenced within multi-sample pools on an Illumina NextSeq 550 with a v2.5 high output kit (75 cycle). The read structure was paired end with Read 1 starting from a custom read 1 primer containing 20 bases with a 12 bp cell barcode and 8 bp UMI, and Read 2 being 50 bases containing transcript information. Sequencing read alignment was performed using version 2.1.0 of the Dropseq pipeline previously described^68^. For each sequencing run, raw sequencing reads were converted from bcl files to FASTQs using bcl2fastq based on Nextera N700 indices that corresponded to individual samples. Demultiplexed FASTQs were then aligned to the mm10 (murine) or hg19 (human) genome using STAR and the DropSeq pipeline on a cloud-computing platform maintained by the Broad Institute. Individual reads were tagged with a 12 bp barcode and 8 bp UMI contained in Read 1 of each sequencing fragment. Following alignment, reads were grouped by the 12 bp cell barcodes and subsequently collapsed by the 8 bp UMI for digital gene expression (DGE) matrix extraction and generation. Cell hashing FASTQs were processed with CITE-seq-Count (v1.4.2, https://zenodo.org/record/2590196) to obtain UMI-collapsed hashing DGE matrices corresponding to the 6 antibody tags.

### Murine scRNA-seq analysis

Before analysis, DGE matrices were pre-processed to remove cellular barcodes with <500 unique genes, >35% of UMIs corresponding to mitochondrial genes, low outliers in standardized house-keeping gene expression^69^, >30,000 UMIs and cellular doublets identified through manual inspection and use of the DoubletFinder algorithm^70^. These pre-processed DGEs are deposited as GEO GSE148524 and are available with interactive visualization tools, metadata and digital gene expression matrices at the Broad Institute’s Single-Cell Portal (https://singlecell.broadinstitute.org) as study SCP1547.

After quality and doublet correction, we performed integrated analysis on a combined dataset of 19,877 cells, with quality metrics for gene number, captured UMIs and percent mitochondrial genes reported in Extended Data Fig. 2. To better control for potential batch effects that may arise in sample handling and library preparation, dimensional reduction and clustering were performed following normalization with regularized negative bionomical regression as implemented in Seurat V3 via SCTransform^71^. We performed variable gene identification and dimensionality reduction utilizing the first 9 principal components based on the elbow method to identify 8 clusters using Louvain clustering (Resolution, 0.45). Following UMAP visualization, we used log-normalized RNA expression for all differential gene expression tests, gene-set enrichment analyses and gene module scoring. Of the 8 original clusters, a single cluster had mixed marker expression corresponding to the secretory goblet and Paneth lineages. Accordingly, we subsetted this cluster and performed variable gene selection and dimensional reduction (14 principal components), and identified 2 previously unreported clusters corresponding to goblet and early secretory cells by Louvain clustering (Resolution, 0.3), which were annotated accordingly in the full dataset. We identified genes enriched across clusters using the Wilcoxon rank sum test, with genes expressed in at least 20% of cells and with a minimum log fold change of 0.5 to identify generic cell types, and corroborated these cell type identities relative to gene signatures coming from an established murine small intestinal scRNA-seq atlas^37^. Gene modules were scored within each cell on the basis of enrichment in gene set expression relative to randomly selected genes of comparable expression levels in each cell^69^, via the AddModuleScore function within Seurat v3. In addition to cell-type module scoring from ref. ^37^, we incorporated gene sets for ISC sub-typing from ref. ^6^, in addition to gene sets representing ISC activity^46^ and genes known to contain NES from the ValidNESs database^39^.

To quantify enrichments in cell populations between treatment and control within the murine dataset, we utilized Fisher’s exact test for each cell type relative to all others at each timepoint. We only considered populations for testing when that cell type accounted for at least 0.5% of cells in both KPT-330 and control samples. We present the relative enrichment or depletion of a cell population with KPT-330 treatment over time as the odds ratio with a corresponding 95% confidence interval, and false discovery rate (FDR)-adjusted *P* values with significance denoted as ‘*’s in corresponding figure legends.

To interrogate differences in signalling pathway activity between cell types and treatment conditions in the organoid differentiation experiment, we employed the PROGENy package^38^ to infer pathway activity across the package’s 14 supported pathways. Pathway activity was inferred on a single-cell basis without permutation and the top 300 genes were used to generate the model matrix, which was appended as a Seurat object assay in accordance with the PROGENy tutorial for scRNA-seq (https://saezlab.github.io/progeny/articles/ProgenySingleCell.html). Pathway activity for the untreated populations is presented as scaled means of pathway activity for each cell type, while Cohen’s *d* was calculated between the single-cell distributions of KPT-330-treated and untreated cells.

To interrogate potential differences in upstream TF activity between cell types and treatment conditions of the organoid differentiation experiment, we employed the DoRothEA package^38^ to infer upstream TF activity in each single cell. Upstream TF activity was inferred on a single-cell basis with the default murine regulon and a minimum of 10 targets per regulon, which was appended as a Seurat object assay in accordance with the DoRothEA tutorial for scRNA-seq (https://saezlab.github.io/dorothea/articles/single_cell_vignette.html). We performed dimensionality reduction on the full DoRothEA assay utilizing the first 7 principal components based on the elbow method to identify 7 clusters using Louvain clustering (Resolution, 0.45). Following UMAP visualization, we used the DoRothEA assay to perform differential upstream TF expression testing, identifying maker TFs for each cluster. To quantify enrichments in upstream TF clusters by cell type between treatment and control, we utilized Fisher’s exact test for each cell type relative to all others for each DoRothEA cluster. We only considered populations for testing when that cell type had at least 10 cells originating from both KPT-330 and control samples within that DoRothEA cluster. We present the relative enrichment or depletion of a cell population with KPT-330 treatment in each DoRothEA cluster as the odds ratio with a corresponding 95% confidence interval, and FDR-adjusted *P* values with significance denoted as ‘*’s in corresponding figure legends.

GSEA was performed on the full rank-ordered list of differentially expressed genes (without fold change or *P-*value cutoffs) using the piano R package^72^ and the MsigDB hallmark v7 gene sets^44,45^. Gene sets with at least 25 and no more than 500 matching genes were considered, and only gene sets with an FDR-corrected *P* < 0.05 were retained.

### Human scRNA-seq analysis

Before analysis, DGE matrices were pre-processed to remove cellular barcodes with <500 unique genes, >35% of UMIs corresponding to mitochondrial genes, low outliers in standardized house-keeping gene expression^69^ and >30,000 UMIs. Antibody hashed arrays were demultiplexed with doublets and negative-staining cells removed following default settings of the Seurat function HTODemux. These pre-processed DGEs are deposited in the Broad Institute Single Cell Portal (https://singlecell.broadinstitute.org) as study SCP1318.

We performed integrated analysis on a combined dataset of 2,484 cells, with quality metrics for gene number, captured UMIs and percent mitochondrial genes reported in Extended Data Fig. 6. Dimensional reduction and clustering were performed following normalization in Seurat V3 via SCTransform^71^. We performed variable gene identification and dimensionality reduction utilizing the first 18 principal components based on the elbow method to identify 7 clusters using Louvain clustering (Resolution, 0.5). Following UMAP visualization, we used log-normalized RNA expression for all differential gene expression tests, gene-set enrichment analyses and gene module scoring. Of the 7 original clusters, a single cluster had mixed marker expression corresponding to the secretory goblet and enteroendocrine lineages. Accordingly, we subsetted this cluster and performed variable gene selection and dimensional reduction (8 principal components), and identified 2 previously unreported clusters corresponding to goblet and enteroendocrine cells by Louvain clustering (Resolution, 0.3), which were annotated accordingly in the full dataset. We identified genes enriched across clusters using the Wilcoxon rank sum test, with genes expressed in at least 10% of cells and with a minimum log fold change of 0.25 to identify cell types, and corroborated these cell type identities relative to known gene markers. Gene modules were scored within each cell on the basis of enrichment in gene set expression relative to randomly selected genes of comparable expression levels in each cell^69^, via the AddModuleScore function within Seurat v3 (for genes known to contain NES, from the ValidNESs database^39^).

To quantify enrichments in cell populations between treatment and control within the human dataset, we utilized Fisher’s exact test for each cell type relative to all others by donor. We only considered populations for testing when that cell type had at least 1 cell in both KPT-330 and control samples. We present the relative enrichment or depletion of a cell population with KPT-330 treatment over time as the odds ratio with a corresponding 95% confidence interval, and FDR-adjusted *P* values with significance denoted as ‘*’s in corresponding figure legends.

To interrogate differences in signalling pathway activity between cell types and treatment conditions in the human organoid experiment, we employed the PROGENy package^38^ to infer pathway activity across the package’s 14 supported pathways. Pathway activity was inferred on a single-cell basis without permutation and the top 300 genes were used to generate the model matrix, which was appended as a Seurat object assay in accordance with the PROGENy tutorial for scRNA-seq (https://saezlab.github.io/progeny/articles/ProgenySingleCell.html). Pathway activity for the untreated populations is presented as scaled means of pathway activity for each cell type.

### Reporting Summary

Further information on research design is available in the Nature Research Reporting Summary linked to this article.

## Supplementary information

Reporting Summary

Supplementary datasets Small-molecule library used in the primary screen, marker genes from scRNA-seq for organoid-differentiation time courses, and differentially expressed genes and gene-set enrichment analysis.

## Source data

SD for Fig. 1 Source data.

SD for Fig. 2 Source data.

SD for Fig. 2 Uncropped blots.

SD for Fig. 3 Source data.

SD for Fig. 4 Source data.

SD for Fig. 5 Source data.

SD for Fig. 6 Source data.

SD for Fig. 7 Source data.

SD for ED Fig. 1 Source data.

SD for ED Fig. 2 Source data.

SD for ED Fig. 2 Uncropped blots.

SD for ED Fig. 3 Source data.

SD for ED Fig. 4 Source data.

SD for ED Fig. 5 Source data.

SD for ED Fig. 6 Source data.

SD for ED Fig. 7 Source data.

## Data Availability

Source data for the figures are provided with this paper. The murine scRNA-seq data are available from the NCBI Gene Expression Omnibus under accession number GSE148524. Interactive visualization tools, metadata and digital gene-expression matrices can be found via the Broad Institute’s Single-Cell Portal (https://singlecell.broadinstitute.org; studies SCP1547 and SCP1318). To protect the genetic information of donors, FASTQ data for the human intestinal organoids are available on request from A.K.S., provided that a data-use agreement can be signed. The ValidNESS database was accessed via http://validness.ym.edu.tw. Source data are provided with this paper.

## Code availability

The analysis scripts for screening and scRNA-seq are available at https://github.com/ShalekLab/Mead_et_al_NatBME_2021.
