# Supplementary material for: Screening for modulators of the cellular composition of gut epithelia via organoid models of intestinal stem cell differentiation
Source: Nat Biomed Eng. 2022 Mar 21;6(4):476–94. doi: 10.1038/s41551-022-00863-9 (PMC9046079; doi:10.1038/s41551-022-00863-9)
Supplement: Supplementary file 1 — Reporting Summary [file 41551_2022_863_MOESM1_ESM.pdf]

nature research

Corresponding author(s): Alex K. Shalek and Jeffrey M. Karp

Last updated by author(s): Sep 16, 2021

Reporting Summary

Nature Research wishes to improve the reproducibility of the work that we publish. This form provides structure for consistency and transparency in reporting. For further information on Nature Research policies, see our Editorial Policies and the Editorial Policy Checklist.

Statistics

For all statistical analyses, confirm that the following items are present in the figure legend, table legend, main text, or Methods section.

n/a	Confirmed	
☐	☒	The exact sample size (*n*) for each experimental group/condition, given as a discrete number and unit of measurement
☐	☒	A statement on whether measurements were taken from distinct samples or whether the same sample was measured repeatedly
☐	☒	The statistical test(s) used AND whether they are one- or two-sided<br>*Only common tests should be described solely by name; describe more complex techniques in the Methods section.*
☐	☒	A description of all covariates tested
☐	☒	A description of any assumptions or corrections, such as tests of normality and adjustment for multiple comparisons
☐	☒	A full description of the statistical parameters including central tendency (e.g. means) or other basic estimates (e.g. regression coefficient) AND variation (e.g. standard deviation) or associated estimates of uncertainty (e.g. confidence intervals)
☐	☒	For null hypothesis testing, the test statistic (e.g. *F*, *t*, *r*) with confidence intervals, effect sizes, degrees of freedom and *P* value noted<br>*Give P values as exact values whenever suitable.*
☒	☐	For Bayesian analysis, information on the choice of priors and Markov chain Monte Carlo settings
☒	☐	For hierarchical and complex designs, identification of the appropriate level for tests and full reporting of outcomes
☐	☒	Estimates of effect sizes (e.g. Cohen's *d*, Pearson's *r*), indicating how they were calculated

Our web collection on statistics for biologists contains articles on many of the points above.

Software and code

Policy information about availability of computer code

Data collection	Single-cell RNA sequencing data were generated using the Illumina NovaSeq 6000 and NextSeq 550. Flow-cytometry data were collected on a BD LSR Fortessa. Fluorescence assay data were collected on a Tecan M1000 Plate Reader.
Data analysis	Sequencing data was de-multiplexed using bcl2fastq v2.20.0.422, and seq-well FASTQs were aligned and processed using the Drop-Seq computational protocol v2.3.0 (https://github.com/broadinstitute/Drop-seq). For antibody hashing experiment, CITE-seq-Count v1.4.2 was used. R v4.0 and associated packages (tidyverse v1.3.0, Seurat v3.1.5, DescTools v0.99.36, scales v1.1.1, ggplot2 v3.3.1, superheat v0.1.0, Matrix v1.2-18, piano v2.4.0, RColorBrewer v1.1-2, viridis v0.5.1, effectsize v0.3.1, crunch v1.26.3, progeny v1.10.0, dorothea v1.0.0, viper v1.22.0) were used to analyse sequencing data. Further analyses were performed using R, Prism for macOS Version 8.3, and FlowJo X Version 10.6.1. Image quantification was performed with Fiji v2.0. Analysis scripts for screening and for single-cell RNA-seq are available at https://github.com/ShalekLab/Mead_et_al_NatBME_2021.

For manuscripts utilizing custom algorithms or software that are central to the research but not yet described in published literature, software must be made available to editors and reviewers. We strongly encourage code deposition in a community repository (e.g. GitHub). See the Nature Research guidelines for submitting code & software for further information.

Data

Policy information about availability of data

All manuscripts must include a data availability statement. This statement should provide the following information, where applicable:

- Accession codes, unique identifiers, or web links for publicly available datasets
- A list of figures that have associated raw data
- A description of any restrictions on data availability

Source data for the figures are provided with this paper. The accession number for the murine single-cell RNA-sequencing data is available from the NCBI Gene

Expression Omnibus under accession number GSE148524. Interactive visualization tools, metadata and digital gene-expression matrices can be found via the Broad Institute's Single-Cell Portal (https://singlecell.broadinstitute.org; studies SCP1547 and SCP1318). To protect the genetic information of donors, FASTQ data for the human intestinal organoids is available on request from A.S., provided that a data-use agreement can be signed. The ValidNESS database was accessed via http://validness.ym.edu.tw.

Field-specific reporting

Please select the one below that is the best fit for your research. If you are not sure, read the appropriate sections before making your selection.

☒ Life sciences ☐ Behavioural & social sciences ☐ Ecological, evolutionary & environmental sciences

For a reference copy of the document with all sections, see nature.com/documents/nr-reporting-summary-flat.pdf

Life sciences study design

All studies must disclose on these points even when the disclosure is negative.

Sample size	For the animal-model analyses, we performed a power analysis to estimate the appropriate number of animals. No statistical tests were used to determine sample sizes for other experiments; sample size was determined from prior experience and from pilot experiments, and at least three biological replicates were used for each experiment (except for single-cell RNA sequencing).
Data exclusions	For PAS+ goblet-cell counting, histological samples that included < 15 countable villi were excluded. PAS+ cell-number variation was large, and it was difficult to collect many countable villi per sample; hence, the point of compromise was 15 (this exceeded the recommended count number, in consultation with a pathologist). Otherwise, no data were excluded from the analyses.
Replication	All experiments were performed in at least three independent biological replicates, except for single-cell RNA sequencing, where a single sample contained single cells derived from thousands of organoids of identical condition. Where applicable, all attempts at replication were successful.
Randomization	For the animal study, mice were randomly allocated to each group. For the cell-based analyses and screening, wells were randomly assigned for each treatment.
Blinding	For histological analyses, the slides were blinded before counting. Otherwise, the samples were not blinded; they were randomized for practical expediency.

Reporting for specific materials, systems and methods

We require information from authors about some types of materials, experimental systems and methods used in many studies. Here, indicate whether each material, system or method listed is relevant to your study. If you are not sure if a list item applies to your research, read the appropriate section before selecting a response.

Materials & experimental systems

n/a	Involved in the study	
☐	☒	Antibodies
☒	☐	Eukaryotic cell lines
☒	☐	Palaeontology and archaeology
☐	☒	Animals and other organisms
☐	☒	Human research participants
☒	☐	Clinical data
☒	☐	Dual use research of concern

Methods

n/a	Involved in the study	
☒	☐	ChIP-seq
☐	☒	Flow cytometry
☒	☐	MRI-based neuroimaging

Antibodies

Antibodies used	FITC-conjugated anti-lysozyme antibody (Dako, F0372), APC-conjugated anti-CD24 antibody (Biolegend, #138505) , anti-lysozyme antibody (Abcam, ab108508), HRP-linked anti-rabbit IgG antibody (Cell Signaling, #7074), anti-Ki67 antibody (BD Biosciences, #550609), anti-Olfm4 antibody (Cell Signaling, #39141), anti-Lysozyme (Thermo Fisher Scientific, RB-372-A), anti-E-cadherin (Thermo Fisher Scientific, 13-1900), anti-Rat IgG (H+L) Highly Cross-Adsorbed Secondary Antibody, Alexa Fluor 488 (Thermo Fisher Scientific, A21208), anti-Rabbit IgG (H+L) Highly Cross-Adsorbed Secondary Antibody, Alexa Fluor 568 (Thermo Fisher Scientific, A10042).
Validation	FITC-conjugated anti-lysozyme antibody (Dako, F0372) and anti-CD24 antibody (Biolegend, #138505) were validated in some previous studies such as BMC Biology 16, 62 (2018).<br><br>anti-Lysozyme (Thermo Fisher Scientific, RB-372-A) was validated in some previous studies such as Cell, 178, 5, 1115, 2019 and by the manufacturer (https://www.fishersci.se/shop/products/lab-vision-lysozyme-muramidase-ab-1-rabbit-polyclonal-antibody-bsa-azide/12603267?searchHijack=true&searchTerm=12603267&searchType=RAPID&matchedCatNo=12603267).

anti-E-cadherin (Thermo Fisher Scientific, #13-1900) was validated in some previous studies such as J Cell Biol., 204, 839, 2014 and by the manufacturer (https://www.thermofisher.com/antibody/product/E-cadherin-Antibody-clone-ECCD-2-Monoclonal/13-1900).

anti-Rat IgG (H+L) Highly Cross-Adsorbed Secondary Antibody, Alexa Fluor 488 (Thermo Fisher Scientific, A21208) and anti-Rabbit IgG (H+L) Highly Cross-Adsorbed Secondary Antibody, Alexa Fluor 568 (Thermo Fisher Scientific, A10042) were validated in many previous studies and by the manufacturer (https://www.thermofisher.com/antibody/product/Donkey-anti-Rat-IgG-H-L-Highly-Cross-Adsorbed-Secondary-Antibody-Polyclonal/A-21208 and https://www.thermofisher.com/antibody/product/Donkey-anti-Rabbit-IgG-H-L-Highly-Cross-Adsorbed-Secondary-Antibody-Polyclonal/A10042).

anti-lysozyme antibody (Abcam, ab108508) was validated in some previous studies such as Nature Communications 11, 37 (2020) and by the manufacturer (https://www.abcam.co.jp/lysozyme-antibody-epr29942-ab108508.html).

anti-Ki67 antibody (BD Biosciences, #550609) was validated in some previous studies such as Nature Communications 9, 485 (2018) and by the manufacturer (https://www.bdbiosciences.com/us/applications/research/intracellular-flow/intracellular-antibodies-and-isotype-controls/anti-rat-antibodies/purified-mouse-anti-ki-67-b56/p/550609).

anti-Olfm4 antibody (Cell Signaling, #39141) was validated in some previous studies such as Cell Reports 24, 2312-2328.e7 (2018) and by the manufacturer (https://www.cellsignal.jp/products/primary-antibodies/olfm4-d6y5a-xp-rabbit-mab-mouse-specific/39141?_=1597801405085&Ntt=39141&tahead=true).

Animals and other organisms

Policy information about studies involving animals; ARRIVE guidelines recommended for reporting animal research

Laboratory animals	8–10-week-old wild type C57BL/6NCrl male mice (#027) were purchased from Charles River. The mice were housed under 12-h light/dark cycles, and provided food and water ad libitum. They were kept at 20–22 °C and in a 30–70% humidity environment.
Wild animals	The study did not involve wild animals.
Field-collected samples	The study did not involve samples collected from the field.
Ethics oversight	All animal studies were approved by the Committee on Animal Care (CAC) at Massachusetts Institute of Technology.

Note that full information on the approval of the study protocol must also be provided in the manuscript.

Human research participants

Policy information about studies involving human research participants

Population characteristics	De-identified adult duodenal tissue was collected during bulk surgical resection and distributed as excess surgical tissue via MGH pathology. Donors were of both sexes, aged 58–74 years old, with pathologies presumed to have no impact on the duodenum.
Recruitment	Small intestinal crypts were isolated from de-identified human bulk surgical resections.
Ethics oversight	All studies were performed under protocols approved by the Massachusetts Institute of Technology (MIT) Committee on the Use of Humans as Experimental Subjects, and excess surgical tissue was collected under Mass General Brigham Protocol 2010P000632, in accordance with IRB guidance.

Note that full information on the approval of the study protocol must also be provided in the manuscript.

Flow Cytometry

Plots

Confirm that:

☒ The axis labels state the marker and fluorochrome used (e.g. CD4-FITC).

☒ The axis scales are clearly visible. Include numbers along axes only for bottom left plot of group (a 'group' is an analysis of identical markers).

☒ All plots are contour plots with outliers or pseudocolor plots.

☒ A numerical value for number of cells or percentage (with statistics) is provided.

Methodology

Sample preparation	Organoids were harvested from Matrigel by mechanical disruption in TrypLE Express (Thermo, #12605010) to remove the Matrigel and to dissociate the organoids into single cells. See Methods for details.
Instrument	We used LSR Fortessa (BD; Koch Institute Flow Cytometry Core at MIT).
Software	The data were analysed using FlowJo X v10.6.1 software.

Cell population abundance	Approximately 50% of the objects detected by the flow were identified as single cells, and about 25–60% of the single cells were alive. All live cells were analysed to calculate the Paneth-cell population.
Gating strategy	FSC and SSC were used for selecting single cells, and live cells were identified as Zombie Violet-negative cells. Extended Data Fig. 1f provides details.

☒ Tick this box to confirm that a figure exemplifying the gating strategy is provided in the Supplementary Information.
