# Supplementary figures and images for: Screening for modulators of the cellular composition of gut epithelia via organoid models of intestinal stem cell differentiation
Source: Nat Biomed Eng. 2022 Mar 21;6(4):476–94. doi: 10.1038/s41551-022-00863-9 (PMC9046079; doi:10.1038/s41551-022-00863-9)

## Figure 2 Source Data

2i - LYZ

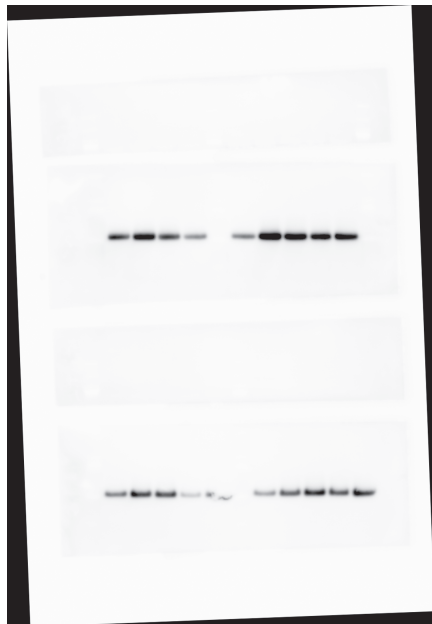

2i - total

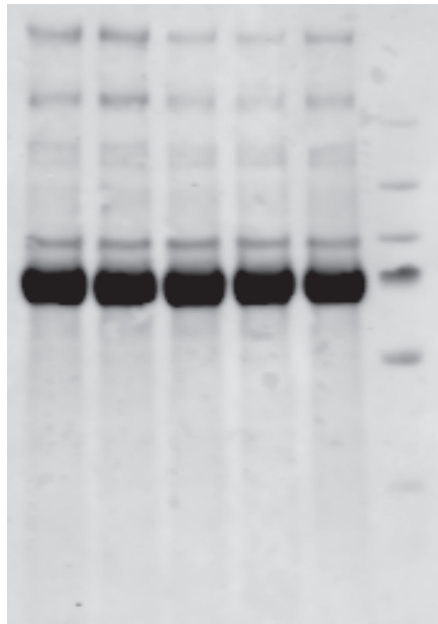

Supplement: Supplementary file 4 — Source data. [file 41551_2022_863_MOESM4_ESM.pdf]

## Extended Data Figure 2 Source Data

2e - LYZ

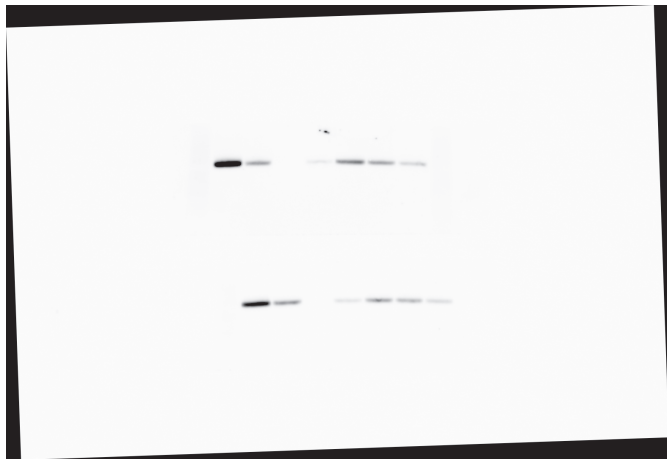

2e - total

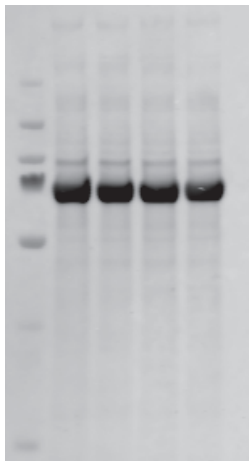

Supplement: Supplementary file 13 — Uncropped blots. [file 41551_2022_863_MOESM13_ESM.pdf]
